# Chalcones as Promising Antitumor Agents by Targeting the p53 Pathway: An Overview and New Insights in Drug-Likeness

**DOI:** 10.3390/molecules26123737

**Published:** 2021-06-19

**Authors:** Joana Moreira, Joana Almeida, Lucília Saraiva, Honorina Cidade, Madalena Pinto

**Affiliations:** 1 Laboratory of Organic and Pharmaceutical Chemistry, Department of Chemical Sciences, Faculty of Pharmacy, University of Porto, Rua de Jorge Viterbo Ferreira 228, 4050-313 Porto, Portugal; up201302558@edu.ff.up.pt; 2 Interdisciplinary Centre of Marine and Environmental Research (CIIMAR), University of Porto, Edifício do Terminal de Cruzeiros do Porto de Leixões, Avenida General Norton de Matos s/n, 4450-208 Matosinhos, Portugal; 3 LAQV/REQUIMTE, Laboratory of Microbiology, Department of Biological Sciences, Faculty of Pharmacy, University of Porto, Rua Jorge Viterbo Ferreira, 228, 4050-313 Porto, Portugal; up201303752@edu.ff.up.pt

**Keywords:** chalcones, antitumor activity, p53, MDM2, drug-likeness, pharmacokinetic properties

## Abstract

The p53 protein is one of the most important tumor suppressors that are frequently inactivated in cancer cells. This inactivation occurs either because the *TP53* gene is mutated or deleted, or due to the p53 protein inhibition by endogenous negative regulators, particularly murine double minute (MDM)2. Therefore, the reestablishment of p53 activity has received great attention concerning the discovery of new cancer therapeutics. Chalcones are naturally occurring compounds widely described as potential antitumor agents through several mechanisms, including those involving the p53 pathway. The inhibitory effect of these compounds in the interaction between p53 and MDM2 has also been recognized, with this effect associated with binding to a subsite of the p53 binding cleft of MDM2. In this work, a literature review of natural and synthetic chalcones and their analogues potentially interfering with p53 pathway is presented. Moreover, in silico studies of drug-likeness of chalcones recognized as p53–MDM2 interaction inhibitors were accomplished considering molecular descriptors, biophysiochemical properties, and pharmacokinetic parameters in comparison with those from p53–MDM2 in clinical trials. With this review, we expect to guide the design of new and more effective chalcones targeting the p53 pathway.

## 1. Introduction

**Figure 1:**
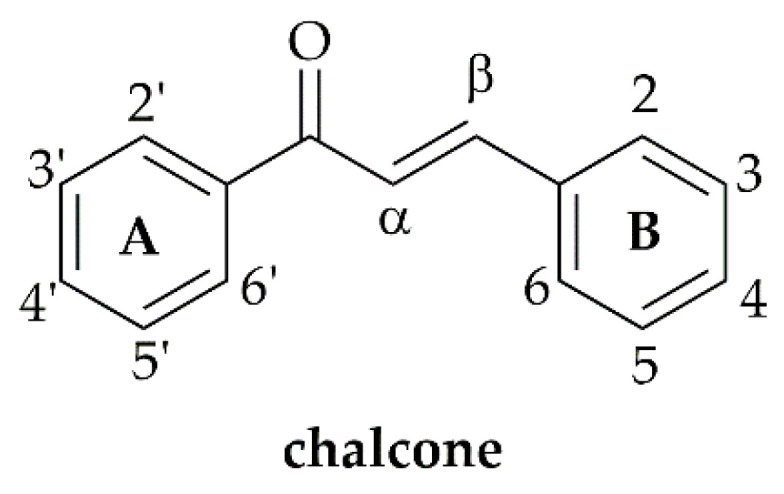
Structure of chalcone.

Chalcones are natural products possessing the common chemical scaffold of a 1,3-diphenyl-2-propen-1-one (Figure 1). These natural products are widely occurring in plants as precursors of other classes of flavonoids, being obtained by a mixed biosynthetic pathway via acetate-malonate and shikimate pathways [1]. Natural chalcones have attracted a large amount of attention from the scientific community due to their broad and interesting biological activities, including anticancer, cancer-preventative, anti-inflammatory, antidiabetic, antioxidant, antimicrobial, and neuroprotective activities, among others [2,3,4,5,6,7,8,9]. 

The antitumor activity of chalcones seems to be mediated by a diverse set of cellular and molecular mechanisms, including the interference with the p53 pathway. The p53 protein is one of the most important tumor suppressors. As a transcription factor, p53 regulates the expression of many pro-apoptotic genes, as well of genes involved in cell cycle arrest, senescence, and DNA repair. In normal cells, which are not under stress conditions, the p53 protein seems to be rather unstable, having a short half-time. As a result, p53 is kept at very low levels and in the inactive state [10]. When cells start to experience stress conditions, p53 becomes active and stable in response to the multiple intra- and extracellular stress signals. When p53 accumulates in the nucleus, it can lead to apoptosis, cell-cycle arrest, and DNA repair [11]. Whether cells are under physiological or stress conditions, the activity and level of p53 are under tight control by diverse mechanisms, including by endogenous negative regulators.

**Figure 2:**
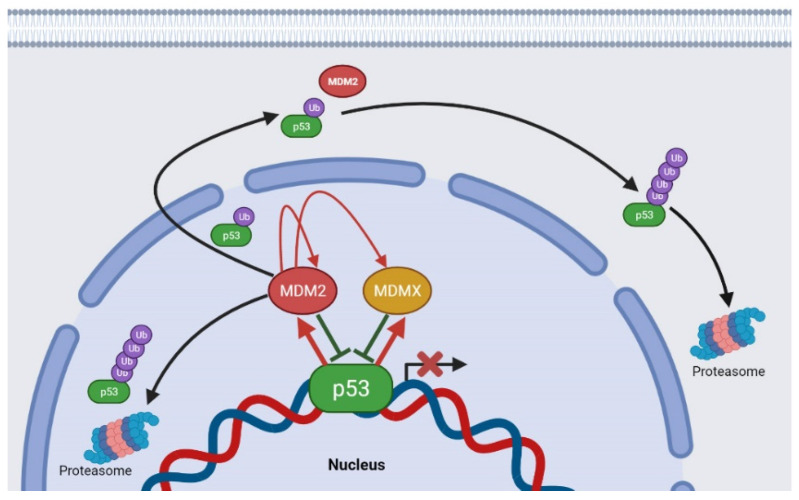
Effects of MDM2 and MDMX in p53. p53 regulates MDM2 and MDMX transcription levels (red arrows), while MDM2 and MDMX inhibit p53 transcriptional function. MDM2 can also promote p53 nuclear export to the cytoplasm and its ubiquitin-mediated degradation by the proteasomes. MDM2 can also promote the nuclear degradation of p53 (polyubiquitination), MDMX ubiquitination, and its own ubiquitination and degradation. Ub: ubiquitin.

Murine double minute (MDM)2 is an E3 ubiquitin-protein ligase and the main endogenous negative regulator of p53 [12]. In normal cells, MDM2 maintains p53 at base levels by regulating its ubiquitination and degradation by the 26S proteasome. When the cell is exposed to stress, MDM2 significantly loses the binding affinity to p53, which results in p53 stabilization [13]. MDM2 is also a transcriptional target of p53, regulating its levels and activity with the creation of a feedback loop, which also regulates its own expression [14]. In addition to its ubiquitination property and proteasomal degradation, MDM2 inhibits the transcription activity of p53 (Figure 2) [15]. In addition to MDM2, MDMX is also implicated in the regulation of p53 activity, as it also inhibits p53 transcriptional function. Nevertheless, MDMX does not exhibit E3 ligase activity and does not directly promote degradation of p53, but it interacts with MDM2, affecting p53 and MDM2 expression levels [16].

**Figure 3:**
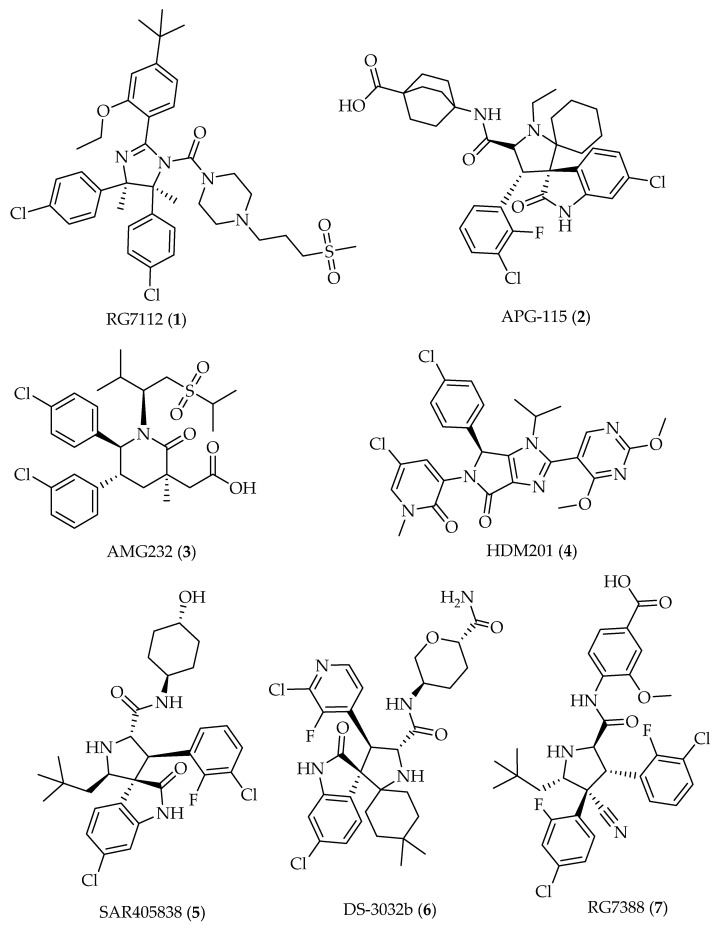
Selected representative small molecules targeting MDM2 in clinical trials.

Accumulating data have shown that wild-type (wt) p53 is a valuable therapeutic target for cancer therapy. In fact, p53 is frequently inactivated in cancer cells, either because the *TP53* gene is mutated or deleted, or because the p53 protein is inhibited by endogenous negative regulators, particularly MDM2. Subsequently, the activation of wt p53 through inhibition of the p53–MDM2 interaction is a promising anticancer strategy. Nevertheless, only a limited number of small-molecule p53-MDM2 inhibitors with superior pharmacokinetic profile have advanced into clinical trials [17] Among these, small molecules **1**–**7** (Figure 3) have shown good pharmacokinetic profile, as well as effective anticancer activity by oral administration in animal models [18,19,20] and in clinical trials [21,22,23,24,25,26,27]. Nevertheless, some adverse effects have been demonstrated, namely, late hematological toxicity and gastrointestinal effects [21,23]. Therefore, new and safe p53-MDM2 inhibitors with potent antitumor activity and adequate drug-like properties are still required as potential anticancer agents.

The present review provides an overview of chalcones with growth inhibitory activity in cancer cells by interfering with the p53 pathway. In addition, chalcone derivatives recognized as p53–MDM2 interaction inhibitors are also reviewed. Considering the importance of prediction of pharmacokinetic properties in the early stages of the drug discovery process to reduce the chance of failure during clinical trials, we also predicted drug-likeness of chalcones reported as p53–MDM2 inhibitors considering molecular descriptors, biophysicochemical properties, and pharmacokinetic parameters in comparison with those of p53–MDM2 inhibitors in clinical trials.

## 2. Interference with p53 Pathway by Natural Chalcones

**Figure 4:**
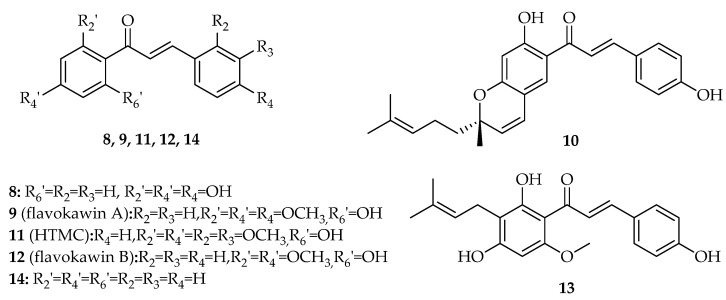
Natural chalcones interfering with p53 pathway.

Several reports have suggested the potential of natural chalcones with different substitution patterns, including hydroxyl, methoxy, and prenyl groups, to interfere with the p53 pathway (Figure 4, Table S1).

The first naturally occurring chalcone reported to induce cell cycle arrest and apoptosis in cancer cell lines, potentially through the interference with p53 pathway, was isoliquiritigenin (**8**, Figure 4) [28,29,30]. When human non-small cell lung cancer A549 cells were treated with 20 µM isoliquiritigenin (**8**), over 46% of cells were arrested in G1 phase, with this percentage increased to 60% with 40 µM of **8** [28]. The protein p53 was upregulated after 6 h of treatment and it reached its maximum after 12 h. Isoliquiritigenin (**8**) also induced apoptosis due to its ability to enhance the expression of p53 downstream targets, specifically Fas and its two ligands, namely, membrane-bound Fas ligand (mFasL) and soluble Fas ligand (sFasL), in A549 cells [28]. The treatment of human hepatoma HepG2 cells with 20 µg/mL of isoliquiritigenin (**8**) also induced G2/S cell cycle arrest and caused DNA fragmentation at 10 µg/mL after 48 h of treatment, which triggered apoptosis. At this concentration, p53 levels also increased after 3 h, reaching maximum levels after 6 h of exposition [29]. In 2009, similar results were also observed for chalcone **8** in human cervical carcinoma HeLa cells. In fact, **8** promoted a potent antiproliferative effect with an IC_50_ value of 9.8 µM, associated with G2/S cell cycle arrest and induction of apoptosis. Furthermore, the authors observed that the exposure of cells to 20 µM isoliquiritigenin (**8**) enhanced the phosphorylation of p53 on residues Ser15 and Ser392. Treatment of HeLa cells with chalcone **8** was also associated with an increase of one of p53 downstream targets, p21 [30]. A more recent work published by Kim et al. (2017) demonstrated that isoliquiritigenin (**8**) generated ROS in human renal carcinoma Caki cells [31]. This, in turn, would stabilize and activate p53, increasing its levels. It also downregulated MDM2, which contributed to the increase of p53 expression [31].

In 2008, a study conducted by Tang et al. showed that flavokawin A (**9**, Figure 4) induced G2/M cell cycle arrest in six mutant p53 papillary bladder cancer cell lines [32]. One of these six lines, human bladder carcinoma HT1197 cells, showed the lowest IC_50_ value of 7.9 µM, and the cell cycle arrest was achieved with a concentration of 40 µM [32]. 

Isolespeol (**10**, Figure 4) is a natural prenylated chalcone isolated by Fang et al. (2008) from the leaves of *Artocarpus communis*, a plant cultivated in tropical and subtropical regions [33]. In 2008, this research team reported that isolespeol (**10**) displayed a promising in vitro growth inhibitory effect, showing an IC_50_ value of 3.8 µM in human liposarcoma SW 872 cancer cells, with this effect associated with increased p53 levels at 3 µM after 3 h exposition [33]. Isolespeol (**10**) also induced apoptosis in SW872 cells through Fas- and mitochondria-mediated pathways [33].

HTMC (**11**, Figure 4) caused reduction of cell proliferation in the lung A549 cancer cell line by 59% at 50 µM. The treatment of A549 cells with 12.5 µM HTMC (**11**) also increased the G1 population of cells from 62.5% to 83.4% and decreased the S population from 31.1% to 13.3% [34]. This cell cycle arrest ability was associated with the effect of **11** on the p21 upregulation, therefore implying p53 upregulation as well, which in turn would inhibit cdc2, causing cell cycle arrest at G1 phase [34].

In 2012, Lin et al. observed that the treatment of human squamous carcinoma KB cells with 5–20 µM of flavokawin B (**12**, Figure 4) induced the upregulation of p21/WAF, p53, and Wee1 [35]. This upregulation resulted in G2/M cell cycle arrest, since p21/WAF can inhibit CDKs and Wee1 prevents the cell from entering into the mitotic stage by catalyzing the inhibitory tyrosine phosphorylation of Cdc2-cyclin B kinase. In addition, there was a decrease in the levels of cyclin A, cyclin B1, Cdc2, and Cdc25C, which contributed to the cell cycle arrest. The apoptotic effects of flavokawin B (**12**) were mainly associated with the decrease in Bcl-2 levels and increase in Bax levels, disrupting the Bcl-2/Bax ratio, as well as the activation of caspases-9, -3, and -8; cleavage of poly ADP ribose polymerase (PARP); and Bid in KB cells [35].

The treatment of lung adenocarcinoma A549 cells with prenylated chalcone xanthohumol (**13**, Figure 4) induced apoptotic cell death and G1 cell cycle arrest [36]. This last event was associated with upregulation of key cell cycle regulators p53 and p21 as well as downregulation of cyclin D1, while the induction of apoptosis was related with the activation of caspase-3 [36].

The effect of unsubstituted chalcone (**14**, Figure 4) was investigated in terms of loss of viability, induction of apoptosis, and alteration of gene expression of p53 and Sp1 in the human osteosarcoma U2OS cell line through in vitro assays [37]. Treatment with chalcone (**14**) induced growth inhibition of these cells in a time- and dose-dependent manner, especially at the concentration of 10 µM after 48 h [37]. It also induced apoptosis and caused Sp1 downregulation at the transcriptional level while upregulating p53 expression at the post-translational level [37]. In 2018, Silva et al. [38] further investigated the effects of chalcone (**14**) on the transcriptomes altered by p53 expression through RNA-Seq analysis [38]. This study was once again performed in the U2OS cell line, which was treated with 50 µM chalcone (**14**) for 24 h. After treatment, the RNAs were isolated and subjected to RNA-Seq. This assay allowed Silva et al. to conclude that the p53 pathway was one of the most highly affected pathways by chalcone (**14**) treatment. Moreover, the *DNAJB1* (*HSP40* family) and *ATF3* genes, which play a role in enhancing stability and activation of p53 by interacting with MDM2, were highly upregulated. This result was further confirmed by RT-PCR and Western blot. Moreover, in order to evaluate the ability of chalcone (**14**) to modify the expression of these two genes in other types of cancer cells, namely, colorectal carcinoma HCT116, pharyngeal squamous-cell carcinoma FaDu, and osteosarcoma SJSA1, we treated cells in the same way as the U2OS cells. The *DNAJB1* gene was upregulated in all cells, and the *ATF3* gene was upregulated in the HCT116 and FaDu, but not in the SJSA1 cells. These results may suggest that the *DNAJB1* gene might be a target of chalcone (**14**) [38]. In 2021, the antiproliferative effect of chalcone (**14**) was studied in human hepatoma HuH7.5 cancer cell line [39]. The results showed an effective dose- and time-dependent cytotoxic effect (IC_50_ = 23.66 μM) without causing toxicity to non-cancerous cells, such as erythrocytes. Chalcone treatment also caused mitochondrial membrane damage and G0/G1 cell cycle arrest, increasing p53 expression and decreasing β-catenin levels. Additionally, chalcone (**14**) decreased the metastatic capacity of HuH7.5, which affected the migration/invasion of this type of cell [39].

## 3. Interference with p53 Pathway by Synthetic Chalcones

Several synthetic chalcone derivatives have been reported for their potential antitumor activity by the interference with p53 pathway. Data concerning structures and results of biological activity are presented as follows into structure subclasses and by chronological order of the year in which they were first reported.

### 3.1. Chalcones with Phenyl Groups

**Figure 5:**
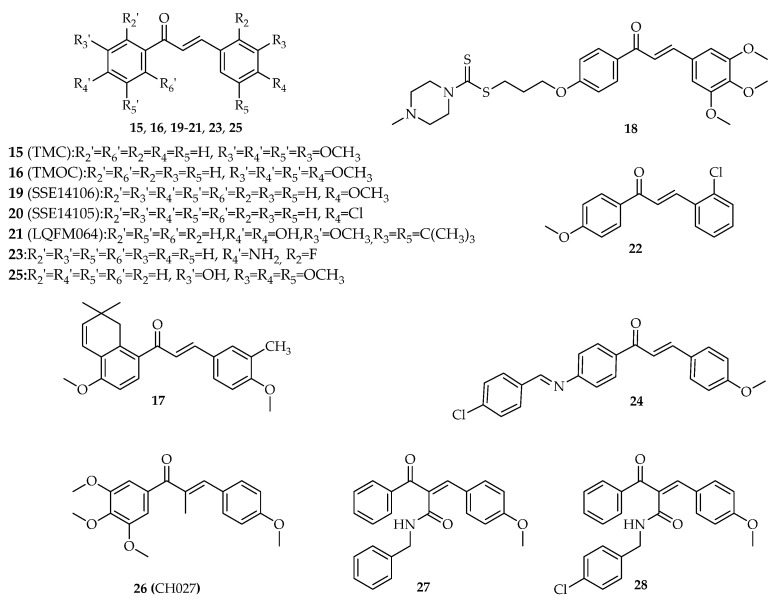
Structure of chalcones **15**–**28**.

More than 10 synthetic chalcones with phenyl groups as A and B rings, presenting different substitution pattern, including hydroxyl, methoxy, halogens and prenyl groups, have demonstrated antiproliferative activity in cancer cell lines by interference with the p53 pathway (Figure 5, Table S2). 

#### 3.1.1. α,β-Non-Substituted Chalcones

In 2014, compound TMC (**15**, Figure 5) was obtained by Claisen–Schmidt condensation using potassium hydroxide as base and ethanol as solvent [40]. TMC (**15**) was identified as a potent inhibitor of cell proliferation, showing IC_50_ values of 3, 4.5, and 1 μM against human oral squamous SCC4, SAS7, and HSC3 carcinoma cells, respectively [40]. Moreover, TMC (**15**) promoted G2/M cell cycle arrest and caused DNA double-strand breaks in SCC4 cells. SCC4 cells treated with 1 µM **15** also revealed increased expression of caspases -3 and -9, PARP, cytochrome *c* release, calpain-1 and -2, phosphorylation of histone H2AX, phosphorylation of checkpoint kinases 2, p53, Bcl-2—antagonist/killer, and Bck-2—associated X protein [40].

In same year, the synthesis of another tetramethoxychalcone (TMOC, **16**, Figure 5) structure related with chalcone **15** was also reported [41]. TMOC (**16**) was identified as an inhibitor of the proliferation and colony formation of cisplatin-sensitive human ovarian A2780 cancer cell line [41]. The treatment of these cells with TMOC (**16**) resulted in G0/G1 cell cycle arrest through downregulation of cyclin D1 and CDK4, as well as upregulation of p16, p21, and p27 proteins. Apoptosis was also induced through suppression of Bcl-2 and Bcl-xL, as well as induction of the expression of Bax and PARP cleavage. It also upregulated p53 [41].

To improve solubility and antitumor activity of the natural chalcone millepachine, researchers obtained a series of derivatives, with SKLB-M8 (**17**, Figure 5) being a potent growth inhibitor of three melanoma cell lines (A2058, CHL-1, and B16F10). SKLB-M8 (**17**) promoted G2/M cell cycle arrest, downregulation of cdc2 expression, and upregulation of p53 in A2058 and CHL-1 cells at 0.1 µM and 0.5 µM, respectively. SKLB-M8 (**17**) also induced apoptosis through downregulation of AKT and phosphorylated mTOR (p-mTOR) [42].

A series of dithiocarbamate-chalcone derivatives with promising antiproliferative activity in three selected cancer cell lines (EC-109, SK-N-SH, and MGC 803) have been reported by Fu et al. (2016) [43], with identified derivative **18** (Figure 5) having the most potent growth inhibitor, showing an IC_50_ value of 2.03 µM in neuroblastoma SK-N-SH cells. The study of the underlying mechanisms involved in the antiproliferative activity of **18** in neuroblastoma SK-N-SH cells revealed that this chalcone dramatically increased the p53 and caspase-3 levels, suggesting the interference with the p53 pathway [43].

A series of 19 chalcones were prepared by reacting 4-substituted acetophenones with appropriate aldehyde in ethanol using ethanolic solution of sodium ethoxide [44]. Most of the synthesized compounds revealed potent antiproliferative activity in HCT116 and breast Cal-51 cancer cell lines. Among tested compounds, 6.25 µM SSE14106 (**19**, Figure 5) and 12.5 µM SSE14105 (**20**, Figure 5), induced accumulation of p53 in HCT116 cells after 4 and 8 h treatment likewise Nutlin-3, a well-known p53–MDM2 interaction inhibitor used as positive control [44]. These results suggested that both compounds can promote the inhibition of p53 degradation pathways. Interestingly, when the activity of **19** with the structure-related 4-hydroxy-dihydrochalcone was compared, the p53 stabilizing activity was completely lost, reinforcing the importance of the α,β-unsaturation to this effect [44].

Cabral et al. (2017) verified that LQFM064 (**21**, Figure 5) induced G0/G1 cell cycle arrest with increased p53 and p21 expressions in breast MCF-7 cancer cells [45]. Following treatment with LQFM064 (**21**), externalization of phosphatidylserine; cytochrome *c* release; increased expression of caspases-7, -8, and -9; reduced mitochondrial membrane potential; and reactive oxygen species (ROS) overproduction were also observed. Moreover, LQFM064 (**21**) increased TNF-R1, Fas-L, and Bax levels and reduced Bcl-2 expression, resulting in apoptosis of these cells [45].

In 2018, Mu et al. tested the antiproliferative activity of compound L6H4 (**22**, Figure 5) on gastric BGC-823 cancer cells [46]. The authors concluded that **22** was able to inhibit cell proliferation by inducing apoptosis and enhancing the expression levels of p53, p21, Bax, and Bcl-2, both in vitro and in vivo. The expression of p53 was increased at 10 µM L6H4 (**22**) after 48 h in in vitro assays. On the other hand, in vivo studies on male BALB/c nude mice showed an increase of p53 levels after treatment with 15 µM L6H4 (**22**) for 48 h [46].

A series of amino-chalcone derivatives were synthesized by Santos et al. (2019) [47]. All the obtained compounds were evaluated for their antiproliferative activity in estrogen receptor-positive (MCF-7) and triple-negative breast cancer (TNBC) cells (MDA-MB-231), with compound 2-fluoro-4′-aminochalcone (**23**, Figure 5) being one of the most active (IC_50_ = 13.2 ± 3.5 µM and IC_50_ = 34.7 ± 5.2 µM, respectively) against both cell lines. Further studies with MCF-7 cells suggested that chalcone **23** upregulated p53 protein expression and did not affect Sp1 protein expression [47].

Salem et al. (2019) synthesized a small library of chalcone derivatives and studied their antiproliferative activity against breast MCF-7 and liver HepG2 cell lines in addition to normal fibroblasts (WI-38) [48]. The antiproliferative activity of the most promising compound (**24**, Figure 5) was associated with the formation of free radicals such as phenoxide radicals, which arrested cell cycle through enhancing the expression of p53 and induced apoptosis by activation of caspases-3 and -9 [48].

In 2020, the new chalcone derivative Lj-1-59 (**25**, Figure 5) was discovered as a promising antitumor agent in different melanoma cell lines (A375, SK-Mel-5, and SK-Mel-28, 1.2 < IC_50_ < 2 µM) [49]. The IC_50_ values obtained for human melanocyte PIG1, normal mouse skin epidermal JB6, normal human skin fibroblast BJ, and normal human heart myoblast H9C2 cell lines (4.2 < IC_50_ < 10 μM) suggested that the cytotoxicity of lj-1-59 (**25**) was selective to melanoma cells. It was found that compound **25** inhibited melanoma cell proliferation in *vitro* and in vivo, induced G2/M cell cycle arrest and promoted apoptosis associated withPARP cleavaged, increased Bax, and decreased Bcl-2 expression levels in SK-Mel-5 and SK-Mel-28 cell lines. Additionally, results revealed that Lj-1-59 (**25**) regulates various pathways, such as DNA replication, p53, apoptosis, and the cell cycle, and significantly increases ROS, leading to DNA toxicity in melanoma cell lines [49].

#### 3.1.2. α-Substituted Chalcones

The compound CH027 (**26**, Figure 5), synthesized through the condensation of 1-(3,4,5-trimethoxyphenyl)propan-1-one with 4-methoxybenzaldehyde, was identified as a potent growth inhibitor of human prostatic carcinoma (LNCaP, C4-2 and 22Rv1) cell lines, with IC_50_ values in nanomolar range [50]. It is noteworthy to mention that chalcone **11** had a 1000-fold increased cytotoxicity compared to its structure related non-α-substituted chalcones (**8**, **9**, and **12**), suggesting that the presence of the methyl group in C-α is important for the activity. Using these prostate cancer cells, researchers demonstrated that the antiproliferative effect of CHO27 (**26**) was associated with cell cycle arrest and caspase-dependent apoptosis, with an upregulation of p53 also observed. Moreover, in vivo studies were performed, and it was verified that CHO27 (**26**) treatment retarded the growth of 22Rv1 xenograft tumors and decreased the final tumor weight by 30%. After one dose of administration (1 mg per mouse, approximately 40 mg/kg body weight), the plasma levels of CHO27 (**26**) achieved a peak of about 850 nM by 1 h, then decreased to over 400 nM by 2 h and to 200 nM by 4 h. CH027 (**26**) was also able to phosphorylate p53 and therefore to upregulate p21^Cip1^ in 22Rv1 xenograft tumors [50].

In 2019, a series of 40 α-substituted chalcones were synthesized and screened for their antiproliferative activity against colorectal HCT116 and breast HCC1954 cancer cell lines [51]. Compounds **27** and **28** (Figure 5) were the most potent with GI_50_ values of 0.63 μM and 0.725 μM in HCC1954 cell line and 0.69 μM and 1.59 μM in HCT116 cell line, respectively. Moreover, both compounds induced G2/M cell cycle arrest and caused apoptotic cell death in HCT116 cells. The compounds also stabilized p53 in a dose-dependent manner in HCT116 cells following 24 h of treatment. Furthermore, **27** and **28** (Figure 5) were able to overcome multidrug resistance in two MDR-1 overexpressing multidrug resistant cell lines [51].

### 3.2. Chalcones with Other Aryl Groups

**Figure 6:**
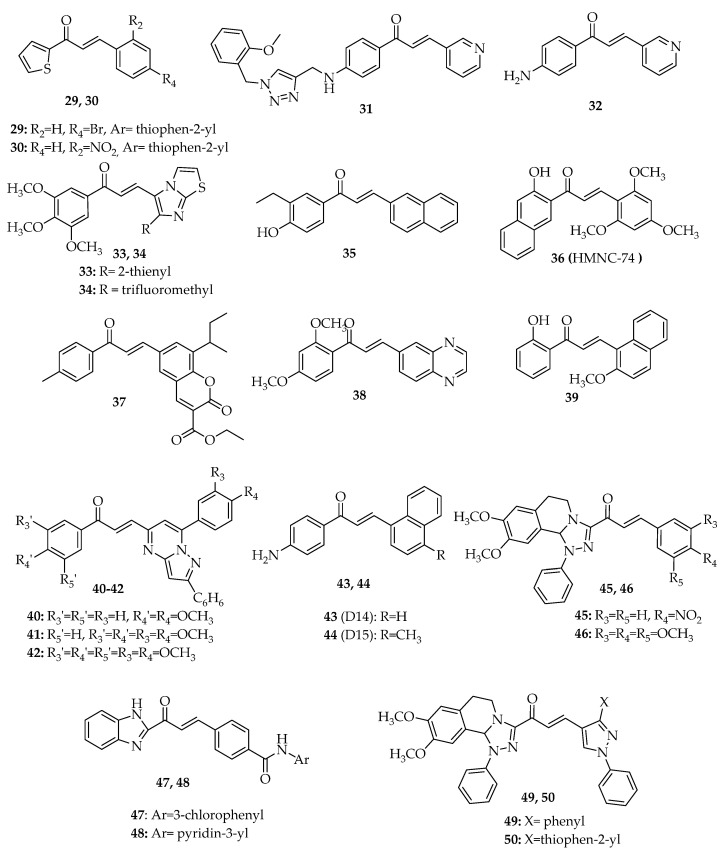
Structure of chalcones **29**–**50**.

In addition to chalcones possessing phenyl groups as A and B rings, several chalcone derivatives with other aryl groups showing promising antiproliferative activity associated with the interference of p53 pathway have also been reported (Figure 6, Table S2). 

#### 3.2.1. Chalcones with Simple Aryl Groups

In 2013, a series of six chalcone derivatives with a 2-acetyl thiophene A ring were synthesized by Claisen–Schmidt condensation between substituted benzaldehyde and 2-acetyl thiophene in the presence of a solution of potassium hydroxide. Their antiproliferative activity was evaluated in colon adenocarcinoma HT-29 cells, with compounds C06 (**29**, Figure 6) and C09 (**30**, Figure 6) being the most promising [52]. Compound **29** upregulated p53 and p21 expression in HT-29 cells, especially at 40 µM, causing G2/M cell cycle arrest. The treatment of these cells with 40 µM **29** was also associated with a marked induction of early or late apoptosis, mainly through the increase of caspase-9 mRNA levels [52]. On the contrary, the structurally related chalcone **29** increased p53 at 40 µM, showing no changes in p21 transcription levels. Like **29**, chalcone **30** also induced a similar late apoptosis and a higher early apoptosis at 40 µM, while revealing a lower cytotoxic effect when compared to C06 (**29**) [52].

Fu et al. (2016) synthesized the 1,2,3-triazole-chalcone hybrid **31** (Figure 6) and tested its effect on SK-N-SH, HepG-2, and human gastric MGC-803 cell lines [53]. Compound **31** showed excellent inhibitory effect against the three tested human cancer cell lines with the IC_50_ value ranging from 1.53 to 2.73 µM [53]. More studies were performed, and it was verified that compound **31** induced G1 cell cycle arrest and apoptosis. This effect was further investigated, and the authors concluded that **31** increased the levels of p53 and caspase-3 while also decreasing the levels of pro-caspase-3 [54]. 

In addition to amino-phenyl-chalcone **26** (Figure 5), Santos et al. (2019) synthesized amino-aryl-chalcone **32** (Figure 6) by Claisen–Schmidt condensation. This compound (**32**) was also reported as one of the most promising regarding its antiproliferative effect against MCF-7 and MDA-MB-231 cell lines (IC_50_ = 15.7 ± 5.9 µM and IC_50_ = 33.9 ± 7.1 µM, respectively) [47]. In MCF-7 cells, chalcone **32** upregulated p53 protein expression [47].

#### 3.2.2. Chalcones with Fused Aryl Groups

Kamal et al. (2010) synthesized a small library of imidazo[2,1-*b*]thiazole chalcone derivatives by Claisen–Schmidt condensation between appropriately substituted acetophenones and imidazo[2,1-*b*]thiazole aldehydes, in the presence of sodium hydroxide. Among the studied compounds, imidazothiazole–chalcone derivatives **33** and **34** (Figure 6) were shown to be the most potent growth inhibitors in a panel of 60 human cancer cell lines. Compound **33** showed a strong cytotoxicity effect, with GI_50_ values ranging from 0.26 to 4.74 µM in a wide range of cancer cell lines, including leukemia (CCRF-CEM, K-562, RPMI-8226, SR), colon (HCC-2998, HCT-15, KM12), prostate (PC-3, DU-145), breast (MCF-7, T-47D, MDA-MB-468), non-small-cell lung (NCI-H23, NCI-H460, NCI-H522), CNS (SF-295, U251), ovarian (IGROV1, NCI/ADR-RES, OVCAR-8), renal (UO- 31), and melanoma (MDA-MB-435, SK-MEL-2, SK-MEL-5) cancer cell lines. Compound **34** had a more modest activity against the SR leukemia and HCT116 colon cancer cell lines, with GI_50_ values of 0.5 µM and 1 µM, respectively. In order to evaluate the effect of these chalcones, the researchers performed flow cytometry analysis at the concentration of 4 µM for 24 h. Compounds **33** and **34** caused G0/G1 cell cycle arrest, with this effect related to the decrease of cyclin D1, A, and E1 levels and the increase of CDK4 levels, as well as the upregulation of p53, p21, p27, and chk2. Moreover, **33** and **34** elicited the characteristic features of apoptosis in MCF-7 cells such as enhancement in the levels of p53, p21, and p27; suppression of NF-κB; and upregulation of caspase-9 [55].

In 2010, Pedrini et al. synthesized a series of 10 chalcones by aldol condensation between 2-naphthaldehyde and substituted acetophenones in the presence of potassium hydroxide as catalyst. A preliminary screening of this series using lymphoblastic leukemia L-1210 cells showed that chalcone **35** (Figure 6) had the most promising cytotoxic effect, with this being associated with induced apoptosis through the increase of Bax, p53, and cleaved caspase-3 expression. Chalcone **35** also caused a significant increase of cells at G0/G1 phase (45.59%) and G2/M phase (42.05%) [56].

In 2014, another naphthalene-chalcone **36** (HMNC-74, Figure 6) was also synthesized by aldol condensation between 2-hydroxy-1-acetonaphthone and 2,4,6-trimethoxybenzaldehyde [57]. This compound (**36**) inhibited the clonogenicity of HCT116 cells and induced G2/M cell cycle arrest and apoptosis. The authors concluded that 10 µM HMNC-74 (**36**) increased p53 levels over time in HCT116 cells expressing wt p53, but not in p53-null HCT116 cells. Moreover, the cleavage of caspase-7 and its substrate PARP was attenuated on p53-null cells, which suggested that the apoptotic effect could be related to p53 activation [57]. In the same year, Lee et al. tested HMNC-74 (**36**) on human colon adenocarcinoma SW620 cell line. Likewise, inhibition of clonogenicity, cell cycle arrest at the G2/M phase, and induction of apoptosis were obtained. The levels of p53 were increased after 12 h of treatment with 10 µM HMNC-74 (**36**), as well as the levels of phosphorylated p53, γ-H2AX, p-Chk2, and Bax [58].

A new coumarin–chalcone hybrid (S009-131, **37**, Figure 6) was synthesized by Singh et al. (2014) [59]. This compound (**37**) revealed promising cytotoxic effect on cervical cancer cell lines (HeLa and C33A, 4.7 < IC_50_ < 7.6 µM). The study of the mechanism underlying the potent antiproliferative activity of compound S009-131 (**37**) in HPV-positive (HeLa) and HPV-negative (C33A) cervical cancer cell lines demonstrated that this chalcone inhibited cell proliferation through apoptosis induction and cell cycle arrest at G2/M phase. Moreover, the pro-apoptotic effect of S009-131 (**37**) was associated with an increase of Bax/Bcl-2 ratio; intracellular ROS; release of cytochrome *c* into the cytosol; and activation of capases-9, -3, and -7. The p53 protein was also upregulated, but only in the C33A cell line, along with its transcriptional target PUMA (in both cell lines), suggesting their role in mediating the apoptotic cell death [59].

Chalcone N9 (**38**, Figure 6) bearing a benzene ring fused with a pyrazine ring, quinoxaline, was reported by Lock-Neckel et al. (2015) [60]. Compound **38** was prepared by aldol condensation between quinoxaline-6-carbaldehyde and 2,4-dimethoxyacetophenone in methanol and potassium hydroxide [60]. Chalcone **38** inhibited cell proliferation and death and caused G1 cell cycle arrest in human glioblastoma U87-MG cell line. In addition, an inhibition of cyclin A and E expression and a decrease in the expression of CDK2, CDK6, and phosphorylation of Rb was observed in N9-U87-MG glioma cells. N9 (**38**) also upregulated p53 and its downstream transcriptional target p21, stabilized p53, and reduced MDM2 levels. Along with the increase in cleaved caspase-9 expression, these effects triggered apoptosis after 24 h of treatment in a dose-dependent manner [60]. Collectively, these results demonstrate that the induction of p53 and p21 proteins, as well as the activation of mitochondrial apoptosis pathway associated with the inhibition of MDM2, is involved in the antitumor activity of N9 (**38**) [60].

In 2016, Shin et al. [61] investigated the mode of action underlying the antitumor activity of the naphtal chalcone derivative HMP (**39**, Figure 6). Results showed that HMP (**39**, Figure 6) promoted a sub-G0/G1 cell cycle arrest and triggered apoptosis in HCT116 cells. Additionally, HMP (**39**) stimulated the cleavage of caspase-7 and its substrate PARP and promoted γH2AX formation, production of ROS, and upregulation of p53 expression. It is noteworthy to mention that HMP-induced caspase-7 activation was not completely abolished in p53-null HCT116 cells, which indicates that the observed cell death could be induced by both p53-dependent and -independent mechanisms [61].

In 2017, Bagul et al. synthesized the chalcone-linked pyrazolo[1,5-*a*]pyrimidines **40**, **41**, and **42** (Figure 6) by Claisen–Schmidt condensation between pyrazolo[1,5-*a*]pyrimidine-5-carbaldehydes and appropriately substituted acetophenones [62]. These compounds (**40**, **41**, and **42**) showed potent growth inhibitory effect in A549, MDA-MB-231, and prostate cancer DU-145 cell lines, with IC_50_ values between 2.6 and 13.7 µM. A549 cells treated with 2 µM of compounds **40**, **41**, and **42** caused G2/M cell cycle arrest of 29.6%, 29.5%, and 33.11%, respectively. These chalcones increased the levels of p53, p21, and Bax and decreased the expression of Bcl-2 in A549 cells, confirming their ability to trigger an apoptotic cell death [62].

D14 (**43**, Figure 6) and D15 (**44**), two 4′-aminochalcones, were studied on the osteosarcoma U2OS and SAOS-2 cell lines by Seba et al. (2018) [63], particularly in terms of anti-metastatic activity. p53 regulates epithelial–mesenchymal transition (EMT), which confers migratory and invasive properties to cancer cells [63]. These authors suggested that D14 (**43**) and D15 (**44**) upregulated p53 protein levels, thus regulating genes involved in EMT and promoting the inhibition of cell migration and invasion [63].

In 2018, Mohamed et al. [64] reported the synthesis of tetrahydro-(1,2,4)triazolo(3,4-*a*) isoquinoline chalcones by Claisen–Schmidt condensation of 3-acetyl-8,9-dimethoxy-1-phenyl-1,5,6,10b-tetrahydro-[1,2,4]triazolo[3,4-*a*]isoquinoline, and substituted benzaldehydes in the presence of potassium hydroxide solution [64]. Among a series of synthesized chalcones, compounds **45** and **46** (Figure 6) displayed the lowest IC_50_ values in four cancer cell lines (MCF7, A549, HCT116, and HepG2), being selected for further studies concerning the elucidation of the mechanism of action. Both compounds induced G1 cell cycle arrest and stimulated apoptosis of MCF-7 cells. Moreover, it was demonstrated that they induce upregulation of Bax, p53, and caspase-3 genes and downregulation of Bcl-2, MMP1, and CDK4 genes, with compound **45** being more effective in gene regulation and apoptosis induction than compound **46** [64].

Wu et al. (2020) synthesized a series of benzimidazole-derived chalcones containing aromatic amide substituent and screened them for their in vitro antitumor activity against HCT116, HepG2, A549, and human lung CRL-5908 cancer cell lines [65]. These authors explored the mechanism of the most promising compounds (**47** and **48**, Figure 6). The results showed that both compounds induced G2/M cell cycle arrest and apoptosis in HCT116 cells. Additionally, both compounds increased the expression levels of p53 and p21 and decreased the protein level of cdc2. Furthermore, when protein–protein interaction between p53 and MDM2 was explored using co-immunoprecipitation (Co-IP) assay, it was observed that compounds **47** and **48** did not interfere with the interaction between MDM2 and p53. On the other hand, the antitumor activity of these compounds showed obvious differences between the wt HCT116 p53^+^/^+^ (1.34 < IC_50_ < 1.63 µM) and null (p53^−^/^−^) isogenic HCT116 cells (44.38 < IC_50_ < 49.70 µM). A preliminary mechanistic study suggested that these compounds act by upregulating the expression of p53 protein in tumor cells without inhibiting the p53-MDM2 interaction [65].

Recently, Mohamed et al. (2021) reported the synthesis of two new tetrahydro-(1,2,4)triazolo(3,4-a) isoquinoline chalcones (**49** and **50**, Figure 6) with promising in vitro growth inhibitory activity [66]. Molecular modeling was performed to predict the mechanism of action of these compounds. In vitro cytotoxicity showed a strong effect against all tested cell lines (MCF7, A459, HepG2, and HCT116, 19.0 < IC_50_ < 184 µg/mL), and low toxic effect against normal human melanocytes (HFB4, 105 < IC_50_ < 259 µg/mL). Real-time PCR demonstrated that the two compounds upregulated the expression levels of Bax, p53, and caspases -3 and -9, and decreased the expression of anti-apoptotic genesBcl-2, CDK4, and MMP1 in A459 cell line. Additionally, chalcones **49** and **50** induced G2/M cell cycle arrest and increased apoptosis. Cytochrome *c* oxidase and vascular endothelial growth factor (VEGF) enzyme activity were detected by ELISA assay. Furthermore, researchers observed changes in the number and morphology of mitochondria, cell shrinkage, increase in the number of cytoplasmic organelles, membrane blebbing, chromatin condensation, and apoptotic bodies [66].

### 3.3. Chalcone Analogues

**Figure 7:**
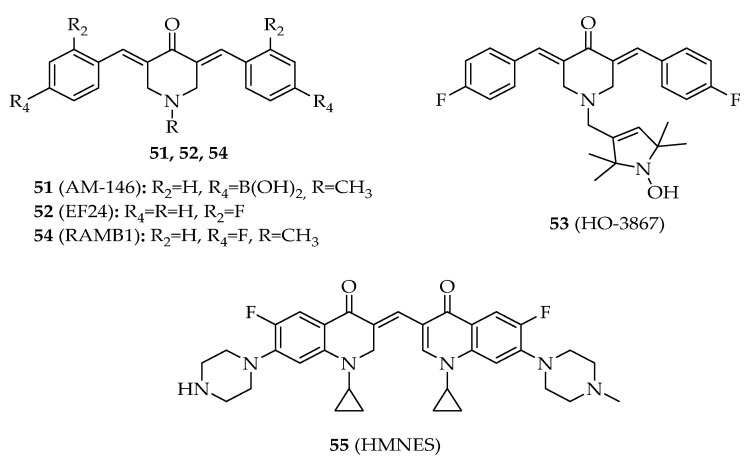
Structure of chalcone analogues **51**–**55**.

In addition to chalcones, five chalcone analogues have been reported to interfere with p53 pathway (Figure 7, Table S2).

In 2006, Modzelewska et al. synthesized a series of chalcone analogues with dienone moiety by Claisen–Schmidt condensation of *N*-methylpiperidine-4-one with appropriate benzaldehydes in the presence of an ethanolic solution of potassium hydroxide [67]. From this series of chalcone analogues, boronic acid derivative AM114 (**51**, Figure 7) was identified as a promising antitumor agent in HCT116 cell line. This compound (**51**) was found to be more cytotoxic to p53 expressing HCT116 cells than p53-null HCT116 cells, suggesting the interference on p53 activity [67]. In that same year, the cytotoxic activity of boronic chalcone analogue **51** was also studied in HCT116 p53^+/+^ cells, showing an IC50 value of 1.49 μM [68]. According to Achanta et al. (2006), the cytotoxic activity of **51** was associated with p53 cellular accumulation. This accumulation seemed to be caused by the inhibition of the 20S proteasome by AM114 (**51**), which in turn leads to the accumulation of ubiquitinated p53 and p21. However, this compound was not able to significantly disrupt the interaction between p53 and MDM2. Curiously, AM114 (**51**) was preferentially toxic to cells expressing wt p53, while the combination of this boronic derivative with ionizing radiation enhanced the cytotoxic activity of the radiation in both wt p53 and p53-null cell. Once again, the chalcone analogue AM114 (**51**) was reported to inhibit deubiquitinating (DUB) enzymes in MDA-MB-231 breast cancer cell line, namely, UCH-L1, UCH-L3, USP2, USP5, and USP8, which are known to regulate the turnover and stability of key regulators of cell survival and proliferation such as p53. The inhibition of these enzymes downregulated several cell cycle promoters, namely, cyclin D1, and upregulated tumor suppressors such as p53, leading to cell cycle arrest and apoptosis [69].

In 2007, the halogenated chalcone analogue with dienone moiety EF24 (**52**, Figure 7) was obtained by allowing *N*-piperidine-4-one to react with a 4-fluorobenzaldehyde under basic aldol condensation conditions [70]. EF24 (**52**) was studied for its ability to inhibit the proliferation of cisplatin (CR)-resistant human ovary cancer cells. EF24 (**52**) increased PTEN expression in human ovarian cancer cells. The upregulation of PTEN resulted in a decrease in Akt and MDM2 and an increase in the expression of p53, also inducing G2/M arrest and apoptosis [70].

Later, Selvendiran et al. (2010) verified that *N*-hydroxypyrroline derivative HO-3867 (**53**, Figure 7) showed a preferential toxicity toward ovarian cancer cells while sparing healthy cells [71]. Studies of mechanism of action were performed, and it was found that compound **53** induced G2/M cell cycle arrest in A2780 cells by modulating several cell cycle regulatory molecules (p53, p21, p27, cyclin-dependent kinase 2, and cyclin) and promoted apoptosis by caspase-8 and -3 activation [71]. In 2011, it was further verified that **53** induced G2/M cell cycle arrest in A2780 cells by modulating the same cell cycle regulatory proteins as Kálai et al. and promoted apoptosis by caspase-8 and -3 activation [71].

In 2011, Anchoori et al. [72] synthesized a series of chalcone analogues known as RAMBs, including RAMB1 (**54**, Figure 7). Treatment of CaSki cervical cancer cells with 10 µM RAMB1 (**54**) led to the accumulation and stabilization of p53. This stabilization caused suppression of cyclin D1 transcription, which indicated that these cells, upon treatment with RAMB1, failed to enter S phase of cell cycle, resulting in loss of viability [72].

In 2016, Ma et al. [73] synthesized the bis-fluoroquinolone chalcone-like derivative HMNE3 (**55**, Figure 7) and assessed its anticancer properties. It caused S-phase cell cycle arrest in human pancreatic adenocarcinoma Capan-1 cell line, which could be related to the inhibition of tyrosine kinase activity. This compound (**55**) also inhibited the ability of topoisomerase II to relax supercoiled pBR322 DNA, which could trigger apoptosis. HMNE3 (**55**) induced an increase in the expression levels of cleaved caspase-9 and -3, upregulated p53 and Bax, and decreased Bcl-2 levels after 48 h of treatment [73].

## 4. Chalcones as Disruptors of the p53–MDM2 Interaction

In addition to chalcones that interfere with p53 pathway, described in Section 2 and Section 3, 25 chalcone derivatives have been proven to interfere with the interaction between p53 and MDM2. Data concerning the structure, results of biological activity by chronological order of the year in which they were first reported, are presented in this section and in Table S3.

**Figure 8:**
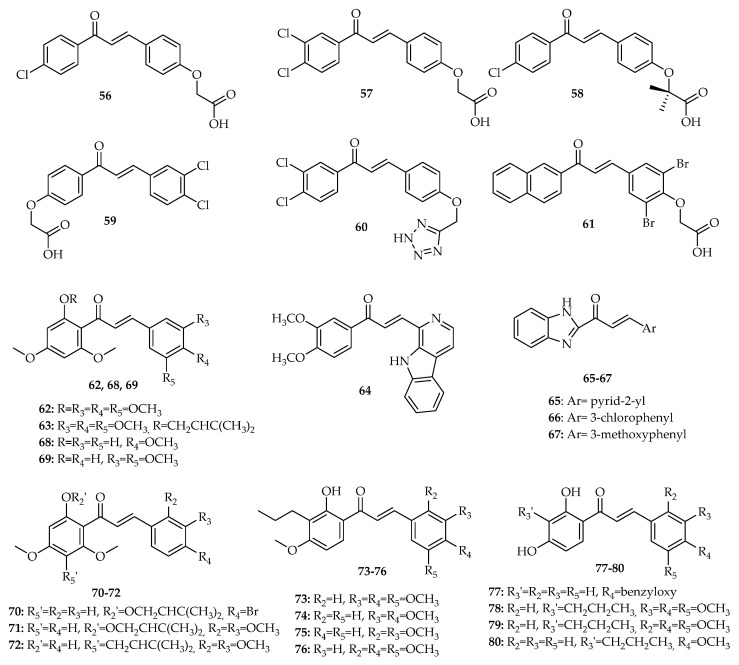
Structures of chalcones **56**–**80**.

The first report about chalcones with p53–MDM2 inhibitory effect was conducted by Stoll et al. (2001) [74]. This research group proved that the chalcone derivatives **56**–**61** (Figure 8) inhibited the MDM2 binding to a p53 peptide using multidimensional NMR spectroscopy and ELISA assay. Compounds **57**, **60**, and **61** denatured MDM2 and compound **59** led to MDM2 aggregation. In addition to this, the authors also tested these compounds for dissociation of preincubated p53–MDM2 complexes and release of p53 active from DNA binding using the full-length p53 protein in an electrophoretic gel mobility shift assay (EMSA). Compounds **56** and **58** inhibited the p53–MDM2 complex, but without releasing p53 [74]. These interactions seemed to influence the p53 protein, which was not predicted from the ELISA test. Compounds **57**, **60**, and **61** partially removed MDM2 from the p53–MDM2 complex [74], while compound **59** completely released active p53 from MDM2 binding. Considering the mentioned studies, some SAR considerations can be proposed. When comparing the results obtained for these compounds, we can infer that the presence of chorobenzene A rings seems to be more favorable for the p53–MDM2 inhibitory activity than the presence of phenoxyacetic acid A ring. Moreover, the presence of 3,4-dichlorobenzene A ring is associated with a higher p53–MDM2 inhibitory effect than a 4-chlorobenzene A ring. Although different substitution on B ring is tolerated, the presence of a phenoxyacetic acid B ring is preferential for this activity.

In 2015, Leão et al. investigated the impact of chalcone **62** (Figure 8) and its prenylated derivative **63** (Figure 8) on the p53–MDM2 interaction using a yeast growth inhibition assay with *Saccharomyces cerevisiae* cells co-expressing human MDM2 and p53 [75]. Their tumor growth-inhibitory effects were assessed on HCT116 cell lines with wt p53 and its p53-null derivative, followed by analysis of cell cycle and apoptosis. The biological results obtained showed that **63** had a more potent growth inhibitory effect than its precursor (**62**), suggesting that the presence of a prenyl group was favorable for the activity. This activity was related to the activation of the p53 pathway through induction of cell cycle arrest and a mitochondria-dependent apoptosis. Through Co-IP assay, it was also demonstrated that 12 µΜ **63** caused a significant decrease of the amount of MDM2 co-immunoprecipitated with p53, confirming the disruption of the p53–MDM2 interaction by this chalcone [75].

In 2016, Singh et al. reported chalcone pyrido[b]indole CPI-7c (**64**, Figure 8) as a promising antitumor agent in A549, MCF-7 colorectal adenocarcinoma DLD-1 and SW-620, and ovarian adenocarcinoma SKOV-3 cell lines [76]. Results showed that CPI-7c (**64**) strongly upregulated DR4 and DR5 proteins and downregulated the anti-apoptotic protein XIAP. However, it did not induce significant changes in the expression of Bax, Bak, Bcl-2, or cytochrome *c* release. Most importantly, this compound (**64**) was able to promote ubiquitination and degradation of MDM2 by binding directly to its *N*-terminal and RING domains, allowing p53 to be stabilized and activated [76].

In same year, Wu et al. (2016) synthesized a series of novel benzimidazole-2-substituted phenyl or pyridine propyl ketene derivatives and evaluated their antiproliferative activity against HCT116, MCF-7, and HepG2 cell lines [77]. Compounds **65**, **66**, and **67** (Figure 8) have been identified as the most promising compounds (**65**: 0.06 < IC_50_ < 3.6 µM, **66**: 0.03 < IC_50_ < 9.8 µM, and **67**: 0.6 < IC_50_ < 21.7 µM). Mechanistic studies concerning the activity of these compounds suggested that the antiproliferative effect was p53-dependent in vitro. These compounds showed a superior potency in HCT116 p53^+/+^ cells (IC_50_: 0.04 < IC_50_ < 0.8 µM) compared with HCT116 p53^−/−^ cells (1.5 < IC_50_ < 5.6 µM). It was also found that compounds **65**, **66**, and **67** promoted G1 cell cycle arrest in HCT116 (p53^+/+^) cells. Furthermore, by Co-IP assay, it was also demonstrated that these compounds significantly inhibited the binding of p53 to MDM2. In addition, compounds **65**, **66**, and **67** demonstrated in vivo antitumor activity in BALB/c mice with colon carcinoma HCT116 cells [77].

Inspired by the improvement of the p53-MDM2 inhibitory effect associated with prenylation reported by Leão et al. (2015) [75], Brandão et al. (2018) later synthesized a small library of prenylchalcones and their non-prenylated precursors and evaluated their ability to inhibit the p53–MDM2 interaction using a yeast-based assay [78]. From this work, chalcones **68** and **69** (Figure 8) and prenylated derivatives **70**–**72** (Figure 8) were identified as potential disruptors of p53–MDM2 interaction using a yeast cell-based assay. When the data of yeast-based assay are analyzed, some SAR conclusions can be inferred. Comparing the effect of prenylated chalcones with structure-related non-prenylated precursors, one can see that for most of the derivatives, the prenylation was associated with an improvement of the p53–MDM2 inhibitory effect. For non-prenylated chalcones, it seems that the presence in B ring of 4-methoxy or 3,5-dimethoxy phenyl groups may increase the activity, but the presence of 4-bromo or 4-fluorophenyl groups, as well as 2,3- or 3,4-dimethoxy-and 2,3- or 3,5-dichlorophenyl groups, is associated with a decrease of the activity. On the other hand, for prenylated chalcones, the presence in B ring of 4-bromo or 2,3-dimethoxy phenyl groups are favorable for the activity, while the presence of 2,3- or 3,5-dichloro phenyl groups was found to be associated with a decrease of the activity, with this reduction of effect also observed for 4-fluoro prenylated chalcones The capacity of all compounds to inhibit the growth of HCT116 cells was also evaluated, and prenylated chalcone **70**, which was shown to be the most effective as inhibitor of the MDM2 effect on p53 in the yeast assay, was selected to further studies in human cancer cells [78]. Chalcone **70** (Figure 8) showed improved cytotoxicity against human cancer cells expressing wt p53 (HepG2, MCF-7, and A375) cells. In HCT116 cancer cells, it was also shown that the growth inhibitory effect of **70** was associated with the induction of cell cycle arrest, apoptosis, and increased protein expression levels of p53 transcriptional targets (MDM2, p21, GADD45, PUMA, and Bcl-2). Moreover, computational docking studies predicted the interaction of this chalcone with the p53-binding site of MDM2 [78].

The virtual screening of a library of chalcone derivatives led Pereira et al. (2019) to identify potential new MDM2 ligands [79]. The chalcones with the best docking scores obeying the Lipinski rule of five were tested for their potential p53–MDM2 inhibitory effect using a yeast growth-inhibition assay. A total of 20 chalcones were synthetized. It was found that chalcones **73**–**80** (Figure 8) potentially inhibited p53–MDM2 interaction in yeast cells co-expressing p53 and MDM2. From the results obtained for all the synthesized chalcones, some SAR considerations may be undertaken by us. Regarding compounds with dimethoxyphenyl groups as B ring, it seems that the effect of methoxy groups on yeast growth inhibition is dependent on their position. In fact, although the presence of methoxy groups at 2,3- and 3,4-positions are favorable for this activity, the presence of methoxy groups at 2,4- and 3,5-positions are associated with a decrease of activity. When the effects of chalcones with the same B ring (2,4,5- and 3,4,5-trimethoxylated) and a different A ring are compared, it seems that the presence of a propyl chain at C-3′ is crucial for the activity, as well as the fact that it is possible to replace a methoxy by a hydroxyl group at position 4′. Among the compounds **73**–**80**, chalcones **73** and **74** were further studied in non-small cell lung cancer NCI-H460 cells. Both chalcones (**73** and **74**) displayed a pronounced tumor cell growth inhibitory effect, causing cell cycle arrest; induction of apoptosis; and increased protein levels of p53, p21, and PUMA in NCI-H460 cells. Computational docking studies allowed for the prediction that, like Nutlin-3a, chalcones **73** and **74** bind to the p53-binding site of MDM2 [79].

## 5. Prediction of Drug-likeness and Comparison with Reported Compounds Targeting MDM2 in Clinical Trials

An important aspect to be considered in the early stage of drug discovery is the drug-likeness of compounds, which should present appropriate absorption, distribution, metabolism, and excretion (ADME) properties to allow its progression from pre-clinical to clinical evaluation. Early prediction of these parameters in the discovery phase drastically reduces the fraction of pharmacokinetics-related failure in the clinical phases [80]. Therefore, we also predict the drug-likeness of chalcones reported as p53–MDM2 inhibitors considering molecular descriptors, biophysicochemical properties, and pharmacokinetic parameters in comparison with those from p53–MDM2 inhibitors in clinical trials [17], namely, RG7112 (**1**) [81], APG-115 (**2**) [19], AMG-232 (**3**) [20], HDM201 (**4**) [24], SAR405838 (**5**) [82], DS-3032b (**6**) [83], and RG7388 (**7**) [26] (Figure 3). The molecular descriptors, physicochemical properties, and pharmacokinetic properties were evaluated using SwissADME web server (http://www.swissadme.ch/ accessed on 22 March 2021) [84].

### 5.1. Molecular Descriptors and Physicochemical Properties

Molecular descriptors/physicochemical properties of chalcones **56**–**80** reported as disruptors of the p53–MDM2 interaction as well as compounds **1**–**7** targeting MDM2 in clinical trials were evaluated accordingly to the following chemical features: (i) molecular weight (MW); (ii) number of heavy atoms (HA) and number of aromatic heavy atoms (AHA); (iii) unsaturation, inferred by fraction of carbon sp3 (Fsp3); (iv) flexibility, inferred by the number of rotatable bonds (RB); (v) number of hydrogen bond acceptors (HBA) and donors (HBD); (vi) polarity, inferred by polar surface area (PSA); (vii) lipophilicity, inferred by the log *P* using more than one algorithm; and (viii) solubility, inferred by log S using more than one algorithm. The obtained data for molecular descriptors and physicochemical property values are displayed in Tables S4 and S5 (Supplementary Materials), respectively. For the sake of comparison between the chemical space occupied by chalcones **56**–**80** and selected small molecules in clinical trials **1**–**7** reported as disruptors of the p53–MDM2 interaction, the obtained mean values were taken into consideration.

When the presented results in Table S4 are analyzed, the mean MW for p53–MDM2 inhibitors in clinical trials **1**–**7** (613.12 g mol^−1^) is found to be higher than for chalcones **56**–**80** (362.04 g mol^−1^). Considering the range of values preconized by the drug-likeness guidelines (150 < MW < 500), chalcones were in the preconized limits. It is noteworthy to mention that this mean value is in accordance with the mean MW value of synthetic drugs (325.2 g mol^−1^) [85], as well as the mean MW value (313 g mol^−1^) reported for orally bioavailable cancer drugs [86], in contrast with the MW value obtained for compounds **1**–**7.**

The molecular flexibility can be measured by the number of RB and aromatic character, inferred by unsaturation (Fsp^3^) and the fraction of aromatic heavy atoms (Far = AHA/HA). Table S1 displays the obtained values for these molecular descriptors. Chalcones **56**–**80** have mean RB value (6.88) near the number found for compounds **1**–**7** (7.57), showing general synthetic drugs a mean of 5.4 [85]. In turn, the obtained mean Fsp^3^ value for chalcones **56**–**80** (0.18) is lower than the mean Fsp^3^ value found for compounds **1**–**7** (0.45). These results suggest that chalcones respect the values preconized by the drug-likeness guidelines (0 < RB < 9; 0.25 < Fps^3^ < 1), and compounds **1**–**7** do not respect the limits of unsaturation. For molecular descriptor fraction of aromatic heavy atoms (Far, Table S4), defined as the number of AHA divided by the total number of HA, the obtained mean value for chalcones **56**–**80** was 0.53, and 0.46 for compounds **1**–**7**. When this molecular descriptor is considered, differences in the aromatic character between chalcones and small molecules were not observed (0.53 and 0.46, respectively).

The number of HBA, the number of HBD, and PSA of molecule are frequently used to infer molecular polarity. Regarding the mean values of HBA and HBD presented in Table S4, it was observed that both sets of molecules have substantially more acceptors (mean HBA of **1**–**7**: 6.29; mean HBA of **56**–**80**: 4.88) than donors (mean HBD of **1**–**7**: 2.14; mean HBD of **56**–**80**: 1.04), with these values being in agreement with those reported for synthetic drugs (mean HBA of 4.8; mean HBD of 1.6) [85]. When the PSA values are compared (Table S5), chalcones **56**–**80** are found to have lower PSA values than compounds **1**–**7** (mean PSA of 67.28 and 104.50 Å^2^ for chalcones **56**–**80** and compounds **1**–**7**, respectively). These results suggested a relationship between PSA and MW. Both mean PSA values are in accordance with values of drug-likeness guidelines (20 < PSA < 125 Å^2^). Considering that synthetic drugs have a mean of 53.7 Å^2^, chalcones **56**–**80** appear to be more promising compounds [85]. However, the accepted limit of polar surface area is 140 Å^2^, and thus all compounds adhere to this rule. This aspect is important because the negative effect of a high PSA on intestinal absorption is well established [87].

Lipophilicity was inferred by the Log *P* using different methods (Table S5). Among the sets of tested molecules, chalcones **56**–**80** have a lower mean Log *P* value (2.39 < mean Log *P* value < 4.66) than compounds **1**–**7** (4.08 < mean Log *P* value < 5.83). However, the mean Log *P* values differ from method to method, with the ones obtained with MLOGP being the most discrepant for chalcones. Considering the range of values preconized by the drug-likeness guidelines (0 < Log *P* < 5), chalcones **56**–**80** respect preconized limits, but compounds **1**–**7** do not respect them. However, considering that reported synthetic drugs have a mean of 2.4, all tested compounds showed high mean log *P* values [85].

Solubility was evaluated using different methods to determine Log S. In contrast to Log *P*, Log S values obtained are not method dependent, except Log S value obtained with SILICOS-IT for compounds **1**–**7**. For compounds **1**–**7**, the mean Log S values range between −9.56 and −6.40. For chalcones **56**–**80**, the mean Log S values range between −5.88 and −4.88, indicating that chalcones present the best solubility. However, all tested compounds might face problems of solubility, considering that values greater than −4 are acceptable for a drug [86]. Nevertheless, it should be mentioned that these solubility values do not consider the ionization state. A relationship between MW and aqueous solubility: Log S tends to decrease with increasing of MW, independently of the type of scaffold (Figure S1A, Supplementary Materials). The same relationship between Log S and Log *P* is observed, except for compounds **4** and **6** (Figure S1B, Supplementary Materials).

### 5.2. Rule of Drug-Likeness

Using the same web server (SwissADME, http://www.swissadme.ch/ accessed on 22 March 2021) [84], researchers evaluated several drug-likeness rules, namely, Lipinski, Ghose, Veber, Egan, and Muegge rules. The acceptable value of violations for a drug-like molecule is 1. For easier visualization, a colormap (Figure S2) was applied: green for 100% compliance, yellow when 1 violation was found, and orange when 2 or more violations were found, according to the rules of drug-likeness. As expected, the compliance was dependent of the threshold imposed by each rule. Generally, chalcones **56**–**80** appeared to have more drug-likeness than compounds **1**–**7**. Only chalcone **61** presented more than 1 violation for Ghose rule.

### 5.3. Pharmacokinetic Properties

In this step of prediction, GI absorption (high = good absorption), permeation of BBB (No = is not able to permeate the BBB), ability to be a P-gp substrate (No = is not able to be subtract of P-gp), and ability to inhibit one of five major isoforms of CYP450 (CYP1A2, CYPC19, CYP2C9, CYP2C9, CYP3A4; No = is not to inhibit CYP450) were considered (Table S6).

Considering GI absorption, all chalcones **56**–**80** have high probability of being highly absorbed. However, among the remaining compounds (**1**–**7**), only compounds **4** and **5** may be well absorbed. This fact can be attributed to their lower molecular size when compared to selected small molecules in clinical trials (**1**–**7**). Curiously, all chalcones **56**–**80** have high probability of not being subtracts of P-gp efflux pump, in contrast to compounds **1**–**7**, which have been predicted to be subtracts of P-gp. P-gp is commonly expressed in various organs, including the BBB and brush border membrane of the small intestine [88]. The presence of this efflux pump in the brush border membrane of the small intestine may pump out orally absorbed anticancer agents and, hence, decrease the drug’s bioavailability. Therefore, considering the results obtained for the chalcones **56**–**80** for GI absorption and P-gp, we can hypothesize that these compounds could have an interesting bioavailability. Nevertheless, this should be explored in the future. Conversely, when the probability of all compounds to permeate the BBB is analyzed, the majority of chalcones, except chalcones **60**–**63**, **72**, and **78**–**79**, have a good probability of being BBB permeates, with these compounds being the derivatives with lowest PSA values. The combination of the prediction about the effect of chalcones **56**–**80** in P-gp efflux pump and BBB permeation allow us to predict the possibly effect of most of these chalcones in the CNS, which deserve to be validated in the future.

Considering the ability to inhibit isoforms of CYP450, chalcones **58** and **59** seemed to not interfere with all studied isoforms, except **58**, which may inhibit CYP3A4. All the other chalcones were predicted to inhibit at least two of the five major isoforms. Interestingly, among the different isoforms, CYP3A4 was the isoform with the highest probability to be inhibited by all tested compounds, including **1**–**7**, and isoform CYP2C9 has the highest probability to be inhibited by chalcone derivatives **56**–**80**.

## 6. Conclusions

**Figure 9:**
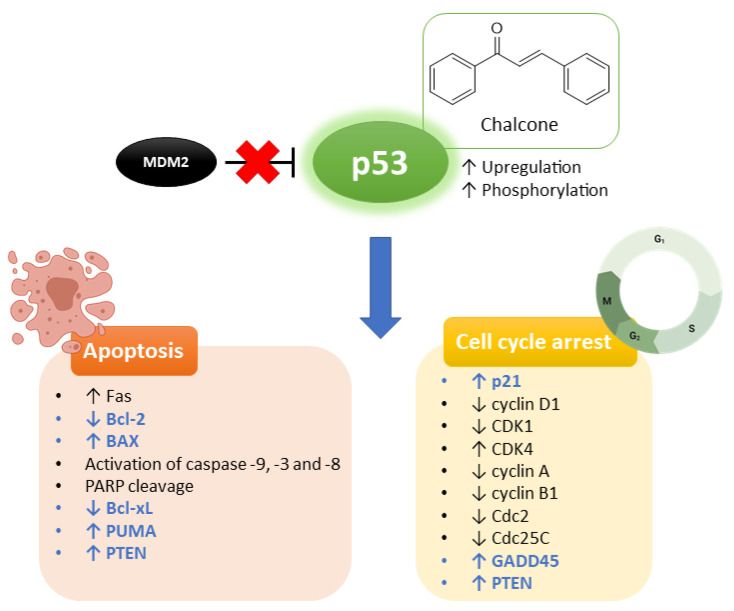
Interference of chalcones with p53 pathway. Chalcones are able to induce changes in the expression levels of several players, ultimately resulting in apoptosis and/or cell cycle arrest. These players can be direct targets of p53 (represented in blue) or indirect targets (represented in black).

The tumor suppressor protein p53 plays an essential role in cell cycle regulation and in the response to numerous stress signals, being one of the most important targets in anticancer therapy. Its inactivation through the interaction with inhibitors, particularly MDM2, is a frequent event in human cancers with wt p53. Therefore, the overexpression of MDM2 will prevent p53 from blocking cell growth and inducing apoptosis. The recovery of this activity by disrupting the interaction between MDM2 and p53 has attracted great attention as a highly selective strategy against tumors. Chalcones are well-known natural products with antiproliferative activity against cancer cells, which seems to be mediated by a diverse set of mechanisms involving the interference with the p53 pathway, as summarized in Figure 9. They are able to block cell cycle progression and induce apoptosis through the interference with the p53 pathway, affecting the expression levels of p53 and increasing the protein expression levels of p53 transcriptional targets. These effects have been reported not only through using in vitro assays with human tumor cell lines, but also with cancer animal models. Although several studies have demonstrated the potential of chalcones as modulators of the p53 pathway, the exact molecular target responsible for the growth inhibitory effect of most of these compounds has not yet been fully clarified. Interestingly, some chalcones have proved to interact with MDM2, being able to dissociate the p53-MDM2 complexes. Nevertheless, the p53–MDM2 inhibitory activity of chalcones has not yet been validated using in vivo assays. Moreover, despite the use of methods showing the direct disruption of the p53–MDM2 interaction (e.g., Co-IP assay, yeast-based assays), crystallography data of co-complex of chalcones with MDM2 have not been reported. Thus, chalcone derivatives were analyzed in terms of docking poses and residues involved in the p53–MDM2 potential interactions. Computational docking studies allowed us to predict that, as with Nutlin-3a, some chalcones bind to the p53-binding site of MDM2. Therefore, they can be considered promising starting points for the structure-based drug design of anticancer therapeutics through the abolishment of the constitutive inhibition of p53 in tumors with overexpressed MDM2. In addition to MDM2, MDMX is an important negative regulator of p53, which can also be overexpressed in wt p53 cancers. Therefore, given the distinct and cooperative function of both MDMs on p53 inactivation and the resistance of MDMX-overexpressing cells to MDM2-only inhibitors, small molecules with dual p53-MDM2/X inhibitory activity represent an ideal strategy for full p53 reactivation. Nevertheless, as far as we know, no chalcone derivatives acting as dual p53-MDM2/X inhibitors have been described. Hence, the study of the effect of chalcones in both endogenous negative regulators MDM2 and MDMX deserves to be explored in the future in order to further the pursuit of the discovery of new p53–MDM2/X dual inhibitors.

Pharmacokinetic studies concerning chalcones with antitumor activity interfering with p53 have received very limited attention, having been reported only for the chalcone derivative CH027 (**26**, Figure 5). Therefore, considering the importance of prediction of pharmacokinetic properties in the early stages of the drug discovery process, we also predict the drug-likeness of chalcones reported as p53–MDM2 inhibitors (**56**–**80**) considering molecular descriptors, biophysicochemical properties, and pharmacokinetic parameters. The obtained values were compared with those from p53–MDM2 in clinical trials (**73**–**79**). The molecular descriptors and physicochemical properties of chalcones **56**–**80** were revealed to be in accordance with the values preconized by the drug-likeness guidelines; however, all tested compounds might face problems of solubility. According to the Lipinski, Ghose, Veber, Egan, and Muegge rules, in general, chalcones **56**–**80** appear to have more drug-likeness than compounds **1**–**7**. Moreover, chalcones **56**–**80** were predicted to have a better GI absorption than most of the tested p53–MDM2 inhibitors in clinical trials and have high probability of not being subtracts of P-gp efflux pump, in contrast to compounds **1**–**7**. Nevertheless, most chalcones, except **60**–**63**, **72**, and **78**–**79**, have good probability of being BBB permeates. This BBB permeation prediction in combination with their possible effect as no subtracts of P-gp efflux pump allow us to envisage the possibility of most of these chalcones to be effective in the CNS, which deserve to be validated in the future.

In general, in silico studies predicted that chalcone derivatives **56**–**80** may have adequate ADME profile. Nevertheless, no pharmacokinetic studies have been performed. Thus, the exploitation of these compounds through in vivo assays should be further explored in the future to validate their value for cancer drug discovery.

We believe that the referred SAR and drug-likeness considerations discussed in this review for chalcones with p53–MDM2 inhibitory effect can pave the way for rational design of new p53–MDM2 inhibitors, leading to accelerate the discovery of more efficient anti-cancer drug candidates in the near future.

## Data Availability

Not applicable.

## Supplementary Materials

The following are available online. Figure S1: Log S vs. molecular weight (MW) of compounds **1**–**7** and chalcones **56**–**80**. Figure S2: Color map of the compliance with the rules of Medicinal Chemistry. Table S1: Natural chalcones with interference in p53 pathway. Table S2: Synthetic chalcones and their analogues with interference in p53 pathway. Table S3: Chalcones as disruptors of the p53–MDM2 interaction. Table S4: Molecular descriptors of compounds reported as disruptors of the p53–MDM2 interaction. Table S5: Physicochemical properties of compounds reported as disruptors of the p53–MDM2 interaction. Table S6: Predictive pharmacokinetic properties compounds reported as disruptors of the p53–MDM2 interaction.

## Abbreviations

ADME	Absorption, distribution, metabolism, excretion
AHA	Aromatic heavy atoms
Akt	Protein kinase B
Bak	Bcl-2 antagonist/Killer
Bax	Bcl-2-associated X protein
BBB	Blood–brain barrier
Bcl-2	B cell lymphoma 2
Bid	BH3-interacting domain death agonist
CNS	Central nervous system
Co-IP	Co-immunoprecipitation
DUB	Deubiquitinating
ELISA	Enzyme-linked immunosorbent assay
EMSA	Electrophoretic gel mobility shift assay
EMT	Epithelial–mesenchymal transition
Far	Fraction of aromatic heavy atoms
Fsp3	Fraction of carbon sp3
HA	Heavy atoms
HBA	Hydrogen bond acceptors
HBD	Hydrogen bond donors
IC_50_	Half-maximal inhibitory concentration
MDM2	Murine double minute 2
MDMX	Murine double minute X
MW	Molecular weight
MW irradiation	Microwave irradiation
NF-kB	Nuclear factor kapa B
PARP	Poly ADP ribose polymerase
p-mTOR	Phosphorylated mTOR
PSA	Polar surface area
PTEN	Phosphatase and tensin homolog
PUMA	p53 upregulated modulator of apoptosis
mFasL	Membrane-bound Fas ligand
RB	Rotatable bonds
ROS	Reactive oxygen species
RT-PCR	Real-time polymerase chain reaction
SAR	Structure–activity relationship
wt	Wild-type
