# Supplementary material for: Chalcones as Promising Antitumor Agents by Targeting the p53 Pathway: An Overview and New Insights in Drug-Likeness
Source: Molecules. 2021 Jun 19;26(12):3737. doi: 10.3390/molecules26123737 (PMC8233907; doi:10.3390/molecules26123737)
Supplement: Supplementary file 1 [file molecules-26-03737-s001.zip › molecules-1221132-supplement.pdf]

molecules

Supplementary Materials

Chalcones as Promising Antitumor Agents by Targeting the p53 Pathway: An Overview and New Insights in Drug-Likeness

Joana Moreira [1,2,†], Joana Almeida [3,†], Lucília Saraiva [3,*], Honorina Cidade [1,2,*] and Madalena Pinto [1,2,*]

1 Laboratory of Organic and Pharmaceutical Chemistry, Department of Chemical Sciences, Faculty of Pharmacy, University of Porto, Rua de Jorge Viterbo Ferreira 228, 4050-313 Porto, Portugal; up201302558@edu.ff.up.pt (J.M.)
2 Interdisciplinary Centre of Marine and Environmental Research (CIIMAR), University of Porto, Edifício do Terminal de Cruzeiros do Porto de Leixões, Avenida General Norton de Matos, S/N, 4450-208 Matosinhos, Portugal
3 LAQV/REQUIMTE, Laboratory of Microbiology, Department of Biological Sciences, Faculty of Pharmacy, University of Porto, Rua Jorge Viterbo Ferreira, 228, 4050-313 Porto, Portugal; up201303752@edu.ff.up.pt (J.A.)
* Correspondence: lucilia.saraiva@ff.up.pt (L.S.); Tel: ++351-220428584 (L.S.); hcidade@ff.up.pt (H.C.); Tel.: +351-220428688 (H.C.); madalena@ff.up.pt (M.P.); Tel.: +351-220428692 (M.P.)
† Authors contributed equally to this work.

Table S1: Natural chalcones with interference in p53 pathway……………………………………………………………….3
Table S2: Synthetic chalcones and their analogues with interference in p53 pathway……………………….………….4
Table S3: Chalcones as disruptors of the p53-MDM2 interaction…….………………………………………………………8
Table S4: Molecular descriptors of compounds reported as disruptors of the p53-MDM2 interaction…..……………...10
Table S5: Physicochemical properties of compounds reported as disruptors of the p53-MDM2 interaction…..……....11
Table S6: Predictive pharmacokinetic properties of compounds reported as disruptors of the p53-MDM2 interaction………..14

Figure S1. (**A**) Log S *vs* molecular weight (MW) of compounds **1–7** and chalcones **56–80**. (**B**) Log S *vs* Log P of compounds **1–7** and chalcones **56-80**. ……………………………………….……………………………………………….13
Figure S2. Color map of the compliance with the rules of Medicinal Chemistry…...………………………………………..13

Table S1. Natural chalcones with interference in p53 pathway.

Chalcones	Cellular/molecular mechanisms	Cell line [Ref]/ Cytotoxic effect (IC_{50})/ p53 activation (Method)
**8**	Increase of p53, Fas-ligand, Fas-receptor, Bax and NOXA expression, p21/WAF1 and Bak levels, and caspase-9 activity<br>Decrease of Bcl-2 and Bcl-X_L, PCNA, MDM2, p-GSK-3β, p-Akt, p-c-Raf and p-PTEN expression levels	A549 [1]<br>IC_{50} = n.d.<br>p53 activity = 20 µM (ELISA)<br>Hep G2 [2]<br>IC_{50} = n.d.<br>p53 activity = 10 µg/mL (ELISA)<br>HeLa [3]<br>IC_{50} = 9.8 µM<br>p53 activity = 10 µM (ELISA)<br>Caki [4]<br>IC_{50} = n.d.<br>p53 activity = 50 µM (Western Blot)
**Flavokawin A (9)**	Increase in p21/WAF1, p27/KIP1, Cdc25C and CDK1 levels<br>Decrease of CDK2, CDK1, Myt1 and Wee1 levels	HT1197 [5]<br>IC_{50} = 7.9 µM<br>p53 activity = 40 µM (Flow Cytometry)
**10**	Increase of p53, Fas, FasL, and Bcl-2 family proteins expression<br>Increase of caspases -3, -8 and -9 expression<br>PARP cleavage<br>Release of cytochrome C	SW 872 [6]<br>IC_{50} = 3.8 µM<br>p53 activity = 5 µM (Western Blot)
**HTMC (11)**	Inhibition of phosphorylation of cdc2 (Tyr15 and Tyr161) and Rb (Ser795 and Ser807/811)<br>Increase of p53 and p21 expression	A549 [7]<br>IC_{50} = 47 µM<br>p53 activity = 6.25 µM (Western Blot)
**Flavokawain B (12)**	Increase in p53, p21/WAF1, Wee1 and Bax levels<br>Activation of caspase-3,-8 and -9<br>PARP and Bid cleavage<br>Decrease of Bcl-2, cyclins A and B1, Cdc2 and Cdc25C levels<br>Release of cytochrome *c*	KB [8]<br>IC_{50} = 30.0 µg/mL<br>p53 activity = 5 µg/mL (Western Blot)
**13**	Suppression of ERK1/2 and p90RSK kinases<br>Inhibition of phosphorylation and activation of the CREB protein<br>Increase in p53 and p21 expression<br>Down-regulation of cyclin D1	A549 [9]<br>IC_{50} = 20.9 µM<br>p53 activity = 10 µM (Western Blot)
**14**	Decrease of Sp1, Sp3 and Sp4 expression<br>Down-regulation of Top2A and MMP-2 transcripts (Sp1 target genes), and MN1<br>Up-regulation of Gadd45A, p21 DNAJB1, ATF3 (p53 target genes), TP53AIP1 and PLK2<br>Increase of p53 expression<br>Decrease of β-catenin expression<br>Cell cycle arrest in G0/G1 phase	U2OS [10]<br>IC_{50} = n.d.<br>p53 activity = 50 µM (Western Blot; RNA-Seq, RT-PCR and Western Blot)<br>HCT-116 [11]<br>IC_{50} = n.d.<br>p53 activity = 50 µM (Western Blot)<br>FaDu [11]<br>IC_{50} = n.d.<br>p53 activity = 50 µM (Western Blot)<br>SJSA1 [11]<br>IC_{50} = n.d.<br>p53 activity = 50 µM (Western Blot)<br>HuH7.5 [12]<br>IC_{50} = 23.66 µM

		p53 activity = 23.66 μM (Immunocytochemical assay)

Table S2. Synthetic chalcones and their analogues with interference in p53 pathway.

Chalcones	Cellular/molecular mechanisms	Cell line [Ref]/ Cytotoxic effect (IC_{50})/ p53 activation (Method)
**Chalcone derivatives with phenyl rings: α, β-nonsubstituted chalcones**		
**15**	Increase of caspase-3 and -9 expressions<br>Increase of PARP, cytochrome c, calpain-1 and -2 expressions<br>Phosphorylation of histone H2AX, checkpoint kinases 2 and of p53	SCC4 [13]<br>IC_{50} = 3 μM<br>p53 activity = 1 μM (Western Blot)
**16**	Increase of Bax expression<br>Decrease of Bcl-2 and Bcl-xL<br>Inhibition of phosphorylation of STAT3 and tyrosine kinases c-Src<br>Down-regulation of cyclin D1 and c-myc<br>Up-regulation of p53 and PTEN	A2780 [14]<br>IC_{50} = 3.5 μM<br>p53 activity = 10 μM (Western Blot)
**SKLB-M8 (17)**	Increase of cleaved Caspase-3<br>Decrease of cleaved procaspase-9 levels and of p-mTOR expression<br>Activation of PARP<br>Down-regulation of AKT and cdc2<br>Up-regulation of cyclin B1 and p53	A2058 [15]<br>IC_{50} = 0.07 μM<br>p53 activity = 0.5 μM (Western Blot)<br>CHL-1 [15]<br>IC_{50} = 0.25 μM<br>P53 activity = 0.5 μM (Western Blot)
**18**	Increase of p53 and caspase-3 levels<br>Pro-caspase 3 cleavage	SK-N-SW [16]<br>IC_{50} = 2.03 μM<br>P53 activity = 5 μM (Western Blot)
**SSE14106 (19)**	Accumulation of p53	HCT116 [17]<br>IC_{50} = n.d.<br>p53 activity = 6.25 μM (Western Blot)
**SSE14105 (20)**	Accumulation of p53	HCT116 [17]<br>IC_{50} = n.d.<br>p53 activity = 12.5 μM (Western Blot)
**21**	Increase of p53, p21 and of caspases -7, -8 and -9 expression and of TNF-R1, Fas-L and Bax levels<br>Externalization of phosphatidylserine<br>Release of cytochrome *c*<br>ROS formation<br>Decrease of Bcl-2 expression	MCF-7 [18]<br>IC_{50} = 21 μM<br>p53 activity = 21 μM (Flow Cytometry)
**22**	Increase of p53, p21, Bax and Bcl-2 expression	BGC-823 [19]<br>IC_{50} = n.d.<br>p53 activity = 10 μM (*in vitro*); 15 μM (*in vivo*) (Western Blot)
**23**	upregulation of p53 expression	MCF-7 [20]<br>IC_{50} = 13.2 ± 3.5 μM<br>p53 activity = 10 μM (Western Blot)
**24**	Formation of free radicals (phenoxide radicals)<br>Increase expression of p53 and caspases-3 and -9	HepG2 [21]<br>IC_{50} = 10.3 ± 1.8 μM<br>p53 activity = 100 nM (qRT-PCR)
**25**	Cell cycle arrest at the G2/M phase<br>ROS formation<br>Induced PARP cleavage	SK-Mel-28 [22]<br>IC_{50} = 1.368 μM<br>p53 activity = 5 μM (RNA-seq)

	Increased of BAX expression<br>Decreased of Bcl-2 expression	
**Chalcone derivatives with phenyl rings: α-substituted chalcones**		
**CH027 (26)**	Increase in p53 activity<br>Up-regulation of p21 ^{Cip1}<br>PARP cleavage	LNCaP [23]<br>IC_{50} = 13 nM<br>p53 activity = 10 nM (Western Blot)<br>22Rv1 [23]<br>IC_{50} = 15 nM<br>p53 activity = 10 nM (Western Blot)
**27**	Cell cycle arrest in S and G2/M phase<br>Activation of PARP<br>Up-regulation of p53	HCC1954 [24]<br>IC_{50} = 0.63 ± 0.06 μM<br>HCT116 [24]<br>IC_{50} = 0.69 ± 0.04 μM<br>p53 activity = 1.56 μM (Western Blot)
**28**	Cell cycle arrest in S and G2/M phase<br>PARP cleavage<br>Up-regulation of p53	HCC1954 [24]<br>IC_{50} = 0.63 ± 0.06 μM<br>HCT116 [24]<br>IC_{50} = 0.69 ± 0.04 μM<br>p53 activity = 1.56 μM (Western Blot)
**Chalcone derivatives with simple aryl ring**		
**29**	Decrease of Bcl-2 expression<br>Increase of Bax expression<br>Up-regulation of caspase-9, p53 and p21<br>Down-regulation of survivin	HT-29 [25]<br>IC_{50} = 13.4 μM<br>p53 activity = 20 μM (RT-PCR)
**30**	Decrease of Bcl-2 expression<br>Increase of Bax expression<br>Up-regulation of caspase-9 and p53<br>Down-regulation of survivin	HT-29 [25]<br>IC_{50} = 19.5 μM<br>p53 activity = 40 μM (RT-PCR)
**31**	Increase of p53 and caspase-3 levels<br>Pro-caspase 3 cleavage	SK-N-SW [26]<br>IC_{50} = 1.53 μM<br>p53 activity = 5 μM (Western Blot)
**32**	upregulation of p53 expression	MCF-7 [20]<br>IC_{50} = 15.7 ± 5.9 μM<br>p53 activity = 10 μM (Western Blot)<br>MDA-MB-231 [20]<br>IC_{50} = 33.9 ± 7.1 μM
**Chalcone derivatives with fused aryl ring**		
**33**	Increase in cyclins D1, A and E1, CDK4 and IKKα levels<br>Decrease in NF-κB<br>Up-regulation of caspase-9, p53, p21, p27 and chk2	MCF-7 [27]<br>IC_{50} = n.d.<br>p53 activity = 4 μM (Western Blot)
**34**	Increase in cyclins D1, A and E1, CDK4 and IKKα levels<br>Decrease in NF-κB<br>Up-regulation of caspase-9, p53, p21, p27 and chk2	MCF-7 [27]<br>IC_{50} = n.d.<br>p53 activity = 4 μM (Western Blot)
**35**	Increase in p53 and Bax expression and in caspase-9 activity	L-1210 [28]<br>IC_{50} = 54 μM<br>p53 activity = 100 μM (Flow Cytometry)
**36**	PARP and caspase-7 cleavage<br>Increase of p53, γ-H2AX, p-Chk2 and Bax levels	HCT116 [29]<br>IC_{50} = n.d.

		p53 activity = 10 μM (Western Blot)<br>SW620 [30]<br>IC_{50} = 14.5 μM<br>P53 activity = 10 μM (Western Blot)
**S009-131 (37)**	Increase of cytochrome c release<br>Increase of Bax, Bak and p53 expression<br>Decrease of Bcl-2 and Bcl-xL expression<br>Increase of p53 expression<br>Cleavage of caspase-7 and -9	C33A [31]<br>IC_{50} = 4.7 ± 1.0 μM<br>p53 activity = 4.7 μM (Western Blot)
**N9 (38)**	Inhibition in the expression of cyclins A and E<br>Decrease of CDK2 and CDK6 expression and of MDM2 levels<br>Rb inactivation<br>ROS formation<br>Increase of Bax, p53 and p21 levels<br>Caspase-9 cleavage	U87-MG [32]<br>IC_{50} = 0.72 μg/mL<br>p53 activity = 0.1 μg/mL (Western Blot)
**HMP (39)**	Caspase-7 cleavage<br>PARP cleavage<br>γ-H2AX formation<br>ROS formation<br>Up-regulation of p53 and Egr-1	HCT116 [33]<br>IC_{50} = n.d.<br>p53 activity = 50 μM (Western Blot)
**40**	Inhibition of EGFR and STAT3 axis<br>Increase of p53, p21 and Bax levels<br>Decrease of Bcl-2 and procaspase-9 levels	A549 [34]<br>IC_{50} = 2.9 ± 0.3 μM<br>p53 activity = 2 μM (RT-PCR, Western Blot)
**41**	Inhibition of EGFR and STAT3 axis<br>Increase of p53, p21 and Bax levels<br>Decrease of Bcl-2 and procaspase-9 levels	A549 [34]<br>IC_{50} = 3.9 ± 0.4 μM<br>p53 activity = 2 μM (RT-PCR, Western Blot)
**42**	Inhibition of EGFR and STAT3 axis<br>Increase of p53, p21 and Bax levels<br>Decrease of Bcl-2 and procaspase-9 levels	A549 [34]<br>IC_{50} = 7.2 ± 0.4 μM<br>p53 activity = 2 μM (RT-PCR, Western Blot)
**43**	Up-regulation of E-cadherin<br>Downregulation of MMP-2 and MMP-9 proteolytic activities, and of vimetin, N-cadherin and β-catenin mRNA levels<br>Decrease of *Slug* gene expression	U2OS [35]<br>IC_{50} = n.d.<br>p53 activity = 27 μM (Western Blot)
**44**	Up-regulation of E-cadherin<br>Downregulation of MMP-2 and MMP-9 proteolytic activities, and of vimetin, N-cadherin and β-catenin mRNA levels<br>Decrease of *Slug* gene expression	U2OS [35]<br>IC_{50} = n.d.<br>p53 activity = 27 μM (Western Blot)
**45**	Up-regulation of Bax, p53 and caspase-3<br>Down-regulation of BCL2, MMP1 and CDK4	MCF-7 [36]<br>IC_{50} = 50.05 μg/mL<br>p53 activity = 50.05 μg/mL (RT-PCR)
**46**	Up-regulation of Bax, p53 and caspase-3<br>Down-regulation of BCL2, MMP1 and CDK4	MCF-7 [36]<br>IC_{50} = 27.15 μg/mL<br>p53 activity = 27.15 μg/mL (RT-PCR)
**47**	Cell cycle arrest in G2/M phase<br>Up-regulation of p53 and p21 expression<br>Decreased of cdc2 levels	HCT116 [37]<br>IC_{50} = 1.34 ± 0.12 μM<br>p53 activity = 5 μM
**48**	Cell cycle arrest in G2/M phase<br>Up-regulation of p53 and p21 expression<br>Decreased of cdc2 levels	HCT116 [37]<br>IC_{50} = 1.63 ± 0.15 μM<br>p53 activity = 5 μM

**49**	Up-regulation of BAX, p53, and caspases -3, and -9 expression<br>Decreased of Bcl-2, CDK4, andMMP1 expression<br>Cell cycle arrest at the G2/M phase<br>Increase of cytochrome c release	A549 [38]<br>IC_{50} = 19 μg/mL<br>p53 activity = 19 μg/mL (RT-PCR)
**50**	Up-regulation of BAX, p53, and caspases -3, and -9 expression<br>Decreased of Bcl-2, CDK4, andMMP1 expression<br>Cell cycle arrest at the G2/M phase<br>Increase of cytochrome c release	A549 [38]<br>IC_{50} = 12 μg/mL<br>p53 activity = 12 μg/mL (RT-PCR)
**Chalcone analogues**		
**AM-146 (51)**	Accumulation of p21<br>Inhibition of 20S proteasome activity<br>Up-regulation of p53, $p27^{Kip1}$ and $p16^{Ink4A}$<br>Suppression of UCH-L1, UCH-L3, USP2, USP5 and USP8 activity	HCT116 [39]<br>IC_{50} = 1.49 μM<br>P53 activity = 1 μM (Western Blot)<br>HCT116 [40]<br>IC_{50} = 2.8 μM<br>P53 activity = 2.8 μM (MTT)<br>MDA MB 231 [41]<br>IC_{50} = 10.71 μM<br>p53 activity = 5 μM (Western Blot)
**EF24 (52)**	Upregulation of PTEN<br>Decrease of Akt<br>Increase of p53 expression	CR [42]<br>IC50 = 0.65 μM<br>p53 activity = 2 μM (Western Blot)
**HO-3867 (53)**	Upregulation of p53 and p21<br>Decrease of cdk2, Cyclin-A, STAT3 (Tyr705) and JAK1 phosphorylation<br>Increase of Fas/CD95<br>Activation of caspase-3 and -8	A2780 [43]<br>IC50 = n.d.<br>p53 activity = 10 μM (Western Blot)
**RAMB1 (54)**	Increase in p53 levels and caspase-3 activity<br>Decrease of cyclin D1 expression<br>PARP cleavage	CaSki [44]<br>IC_{50} = n.d.<br>p53 activity = 2 μM (Western Blot)
**HMNES (55)**	Decrease of tyrosine-kinase activity, Top-IIα and Top IIβ and Bcl-2 expression<br>Caspase-3 and -9 cleavage<br>Up-regulation of p53 and Bax<br>Up-regulation of Bax	Capan-1 [45]<br>IC_{50} = 2.9 μM<br>p53 activity = 1.6 μM (Western Blot)

Table S3. Chalcones as disruptors of the p53-MDM2 interaction

Chalcone	Method [Ref]/<br>Cytotoxic effect (IC_{50}, Cell line)
**56**	ELISA [46]:<br>IC_{50} = 206 μM
**57**	ELISA [46]:<br>IC_{50} = 49 μM
**58**	ELISA [46]:<br>IC_{50} = 250 μM
**59**	ELISA [46]:<br>IC_{50} = 117 μM
**60**	ELISA [46]:<br>IC_{50} = n.d.
**61**	ELISA [46]:<br>IC_{50} = n.d.
**62**	Yeast screening assay [47]<br>20 % reversion of MDM2 effect at 5 μM (% of solvent)<br>GI_{50} = 65 μM (HCT116)
**63**	Yeast screening assay [47]<br>40 % reversion of MDM2 effect at 5 μM (% of solvent)<br>GI_{50} = 4 μM (HCT116)
**CPI-7c (64)**	RT-PCR [48]<br>IC_{50} = n.d.
**65**	Co-IP [49]<br>At 0.1 μM<br>IC_{50} = 0.06 ± 1.45 μM (HCT116)
**66**	Co-IP [49]<br>At 0.1 μM<br>IC_{50} = 0.04 ± 0.02 μM (HCT116)
**67**	Co-IP [49]<br>At 0.5 μM<br>IC_{50} = 0.58 ± 0.11 μM (HCT116)
**68**	Yeast screening assay [50]<br>~95% reversion of MDM2 effect at 10 μM (% of control)<br>GI_{50} = 10.6 μM (HCT116)
**69**	Yeast screening assay [50]<br>~70% reversion of MDM2 effect at 10 μM (% of control)<br>GI_{50} = 50.0 μM (HCT116)
**70**	Yeast screening assay [50]<br>~75% reversion of MDM2 effect at 10 μM (% of control)<br>GI_{50} = 2.1 μM (HCT116)
**71**	Yeast screening assay [50]<br>~80% reversion of MDM2 effect at 10 μM (% of control)<br>GI_{50} = 11.7 μM (HCT116)
**72**	Yeast screening assay [50] :<br>~80% reversion of MDM2 effect at 10 μM (% of control)<br>GI_{50} = 29.5 μM (HCT116)
**73**	Yeast screening assay [51]:<br>79.4 % growth inhibition (% of p53 effect)<br>GI_{50} = 2.6 μM (HCT116 $p53^{+/+}$)<br>GI_{50} = 2.1 μM (NCI-H460)

**74**	Yeast screening assay [51]:<br>97.2 % growth inhibition (% of p53 effect)<br>GI_{50} = 5.9 μM (HCT116 $p53^{+/+}$)<br>GI_{50} = 3.3 μM (NCI-H460)
**75**	Yeast screening assay [51]:<br>63.7 % growth inhibition (% of p53 effect)
**76**	Yeast screening assay [51]:<br>68.8% growth inhibition (% of p53 effect)
**77**	Yeast screening assay [51]:<br>77.9% growth inhibition (% of p53 effect)
**78**	Yeast screening assay [51]:<br>95.2% growth inhibition (% of p53 effect)
**79**	Yeast screening assay [51]:<br>78.3% growth inhibition (% of p53 effect)
**80**	Yeast screening assay [51]:<br>97.0% growth inhibition (% of p53 effect)

Table S4. Molecular descriptors of compounds reported as disruptors of the p53-MDM2 interaction.

Molecule	Formula	MW	Nº HA	Nº AHA	Far	Fsp³	RB	Nº HBA	Nº HBD
**Selected representative small-molecules targeting MDM2 in clinical trials**									
**1**	$C_{38}H_{48}Cl_2N_4O_4S$	727.78	49	18	0.37	0.47	12	6	0
**2**	$C_{34}H_{38}Cl_2FN_3O_4$	642.59	44	12	0.27	0.56	6	6	3
**3**	$C_{28}H_{35}Cl_2NO_5S$	555.41	38	23	0.61	0.27	6	7	0
**4**	$C_{26}H_{24}Cl_2N_6O_4$	562.5	38	12	0.32	0.52	6	5	4
**5**	$C_{29}H_{34}Cl_2FN_3O_3$	618.53	42	12	0.29	0.53	5	7	4
**6**	$C_{30}H_{34}Cl_2FN_5O_4$	616.48	42	18	0.43	0.32	9	8	3
**7**	$C_{31}H_{29}Cl_2F_2N_3O_4$	568.55	37	12	0.32	0.5	9	5	1
**Mean**	-	613.12	41.43	15.29	0.37	0.45	7.57	6.29	2.14
**Chalcones reported as disruptors of the p53-MDM2 interaction**									
**56**	$C_{17}H_{13}ClO_4$	316.74	22	12	0.55	0.06	6	4	1
**57**	$C_{17}H_{12}Cl_2O_4$	351.18	23	12	0.52	0.06	6	4	1
**58**	$C_{19}H_{17}ClO_5$	360.79	25	12	0.48	0.16	6	5	1
**59**	$C_{17}H_{12}Cl_2O_5$	367.18	24	12	0.5	0.06	6	5	1
**60**	$C_{17}H_{12}Cl_2N_4O_2$	375.21	25	17	0.68	0.06	6	5	1
**61**	$C_{21}H_{14}Br_2O_4$	490.14	27	16	0.59	0.05	6	4	1
**62**	$C_{20}H_{22}O_7$	374.38	27	12	0.44	0.25	8	7	1
**63**	$C_{25}H_{30}O_7$	442.5	32	12	0.38	0.32	11	7	0
**64**	$C_{22}H_{18}N_2O_3$	358.39	27	19	0.70	0.09	5	4	1
**65**	$C_{15}H_{11}N_3O$	249.27	19	15	0.79	0	3	3	1
**66**	$C_{16}H_{11}ClN_2O$	282.72	20	15	0.75	0	3	2	1
**67**	$C_{17}H_{14}N_2O_2$	278.31	21	15	0.71	0.06	4	3	1
**68**	$C_{18}H_{18}O_5$	314.33	23	12	0.52	0.17	6	5	1
**69**	$C_{19}H_{20}O_6$	344.36	25	12	0.48	0.21	7	6	1
**70**	$C_{22}H_{23}BrO_4$	431.32	27	12	0.44	0.23	8	4	0
**71**	$C_{24}H_{28}O_6$	412.48	30	12	0.4	0.29	10	6	0
**72**	$C_{24}H_{28}O_6$	412.48	30	12	0.4	0.29	9	6	1
**73**	$C_{22}H_{26}O_6$	386.44	28	12	0.43	0.32	9	6	1
**74**	$C_{21}H_{24}O_5$	356.41	26	12	0.46	0.29	8	5	1
**75**	$C_{21}H_{24}O_5$	356.41	26	12	0.46	0.29	8	5	1
**76**	$C_{22}H_{26}O_6$	386.44	28	12	0.43	0.32	9	6	1
**77**	$C_{22}H_{18}O_4$	346.38	26	18	0.69	0.05	6	4	2
**78**	$C_{21}H_{24}O_6$	372.41	27	12	0.44	0.29	8	6	2
**79**	$C_{21}H_{24}O_6$	372.41	27	12	0.44	0.29	8	6	2
**80**	$C_{19}H_{20}O_4$	312.36	23	12	0.52	0.21	6	4	2
**Mean**	-	362.04	25.52	13.24	0.53	0.18	6.88	4.88	1.04

Table S5. Physicochemical properties of compounds reported as disruptors of the p53-MDM2 interaction.

Compound ID	TPSA	Log P (iLOGP)	Log P (XLOGP3)	Log P (WLOGP)	Log P (MLOGP)	Log P (SILICOS-IT)	Log P (Consensus)	Mean of Log P values	Log S (ESOL)	Log S (Ali)	Log S (SILICOS-IT)	Mean of Log S values
**Selected representative small-molecules targeting MDM2 in clinical trials**												
**1**	90.90	5.40	7.27	7.10	5.36	7.97	6.62	6.62	-8.41	-9.00	-12.13	-9.85
**2**	98.74	3.80	3.57	6.27	5.02	6.40	5.01	5.01	-5.88	-5.33	-9.99	-7.07
**3**	100.13	3.34	5.78	6.76	4.42	5.77	5.21	5.21	-6.65	-7.65	-8.22	-7.51
**4**	104.37	4.45	3.57	3.99	2.53	3.84	3.68	3.68	-5.58	-5.45	-7.92	-6.32
**5**	90.46	4.21	5.26	4.77	4.17	5.48	4.78	4.78	-6.48	-6.91	-9.09	-7.49
**6**	135.44	3.23	3.38	3.43	2.32	4.36	3.34	3.34	-5.69	-5.90	-9.02	-6.87
**7**	111.45	3.96	4.29	7.21	4.75	6.96	5.43	5.43	-6.09	-6.34	-10.56	-7.66
**Mean**	104.50	4.06	4.73	5.65	4.08	5.83	4.87	-	-6.40	-6.65	-9.56	-
**Chalcones reported as disruptors of the p53-MDM2 interaction**												
**56**	63.60	2.51	3.88	3.59	2.78	3.92	3.34	3.34	-4.26	-4.91	-5.07	-4.75
**57**	63.60	2.66	4.58	4.24	3.28	4.58	3.87	3.87	-4.89	-5.64	-5.66	-5.40
**58**	63.60	0.00	3.86	4.25	2.41	4.39	2.98	2.98	-4.47	-4.89	-5.47	-4.94
**59**	63.60	0.00	4.04	4.13	2.45	4.58	3.04	3.04	-4.64	-5.08	-5.66	-5.13
**60**	80.76	2.30	4.00	3.72	3.17	4.58	3.55	3.55	-4.79	-5.40	-6.88	-5.69
**61**	63.60	3.19	5.89	5.62	4.20	5.73	4.92	4.93	-6.63	-7.00	-7.68	-7.10
**62**	83.45	3.36	3.74	3.22	1.14	3.77	3.05	3.05	-4.32	-5.18	-4.96	-4.82
**63**	72.45	4.39	5.02	4.86	2.14	5.65	4.41	4.41	-5.30	-6.28	-6.48	-6.02
**64**	64.21	2.95	4.06	4.52	1.93	5.09	3.71	3.71	-4.81	-5.11	-7.35	-5.76
**65**	58.64	2.18	2.55	2.75	1.18	3.41	2.41	2.41	-3.38	-3.43	-5.1	-3.97
**66**	45.75	2.57	4.22	4	2.79	4.59	3.64	3.64	-4.61	-4.89	-6.08	-5.19
**67**	54.98	2.72	3.56	3.36	1.94	3.99	3.11	3.11	-4.07	-4.4	-5.59	-4.69
**68**	64.99	2.98	3.80	3.21	1.75	3.60	3.07	3.07	-4.17	-4.86	-4.74	-4.59
**69**	74.22	3.54	3.77	3.21	1.44	3.69	3.13	3.13	-4.24	-5.02	-4.85	-4.70
**70**	44.76	4.39	5.79	5.60	3.73	6.04	5.11	5.11	-5.96	-6.50	-6.96	-6.47
**71**	63.22	4.34	5.05	4.85	2.46	5.54	4.45	4.45	-5.21	-6.12	-6.38	-5.90

**72**	74.22	4.30	5.70	4.72	2.46	5.52	4.54	4.54	-5.69	-7.02	-6.07	-6.26
**73**	74.22	4.10	5.11	4.17	2.11	5.03	4.10	4.10	-5.18	-6.41	-6.02	-5.87
**74**	64.99	3.95	5.14	4.16	2.44	4.94	4.13	4.13	-5.10	-6.25	-5.91	-5.75
**75**	64.99	3.71	5.14	4.16	2.44	4.94	4.08	4.08	-5.10	-6.25	-5.91	-5.75
**76**	74.22	4.11	5.11	4.17	2.11	5.03	4.11	4.11	-5.18	-6.41	-6.02	-5.87
**77**	66.76	3.00	5.00	4.31	2.99	4.52	3.96	3.96	-5.25	-6.14	-6.43	-5.94
**78**	85.22	3.61	4.78	3.86	1.89	4.48	3.72	3.72	-4.96	-6.30	-5.33	-5.53
**79**	85.22	3.75	4.78	3.86	1.89	4.48	3.75	3.75	-4.96	-6.30	-5.33	-5.53
**80**	66.76	3.03	4.84	3.85	2.55	4.31	3.71	3.72	-4.82	-5.98	-5.11	-5.30
**Mean**	67.28	3.11	4.54	4.10	2.39	4.66	3.76	-	-4.88	-5.67	-5.88	-

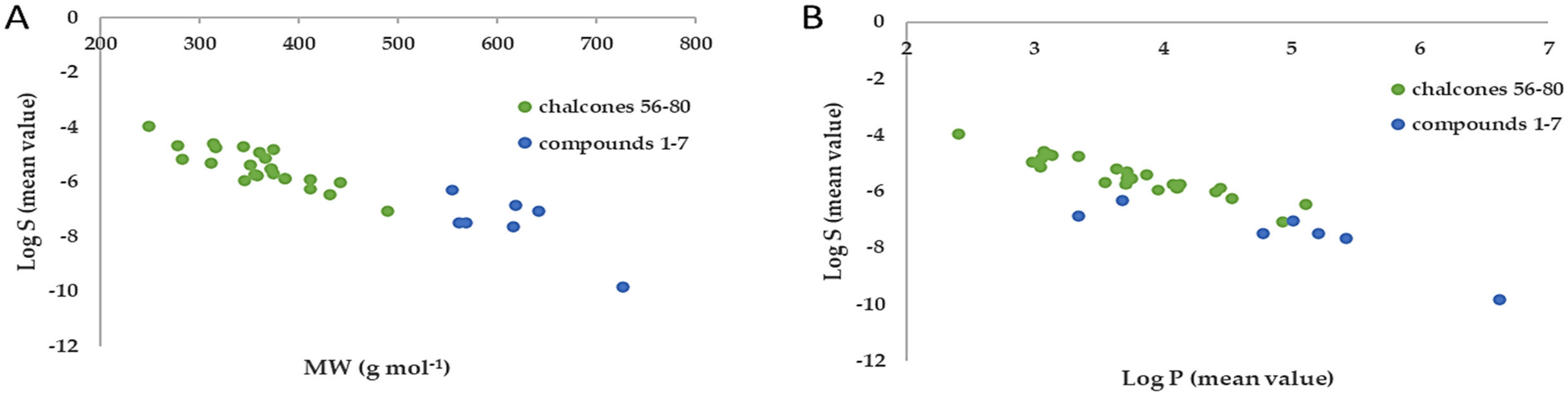

Figure S3. (**A**) Log S *vs* molecular weight (MW) of compounds **1-7** and chalcones **56-80**. For Log S mean values of all tested methods were taken into consideration. (**B**) Log S *vs* Log P of compounds **1-7** and chalcones **56-80**. For Log S and Log P mean values of all tested methods were taken into consideration.

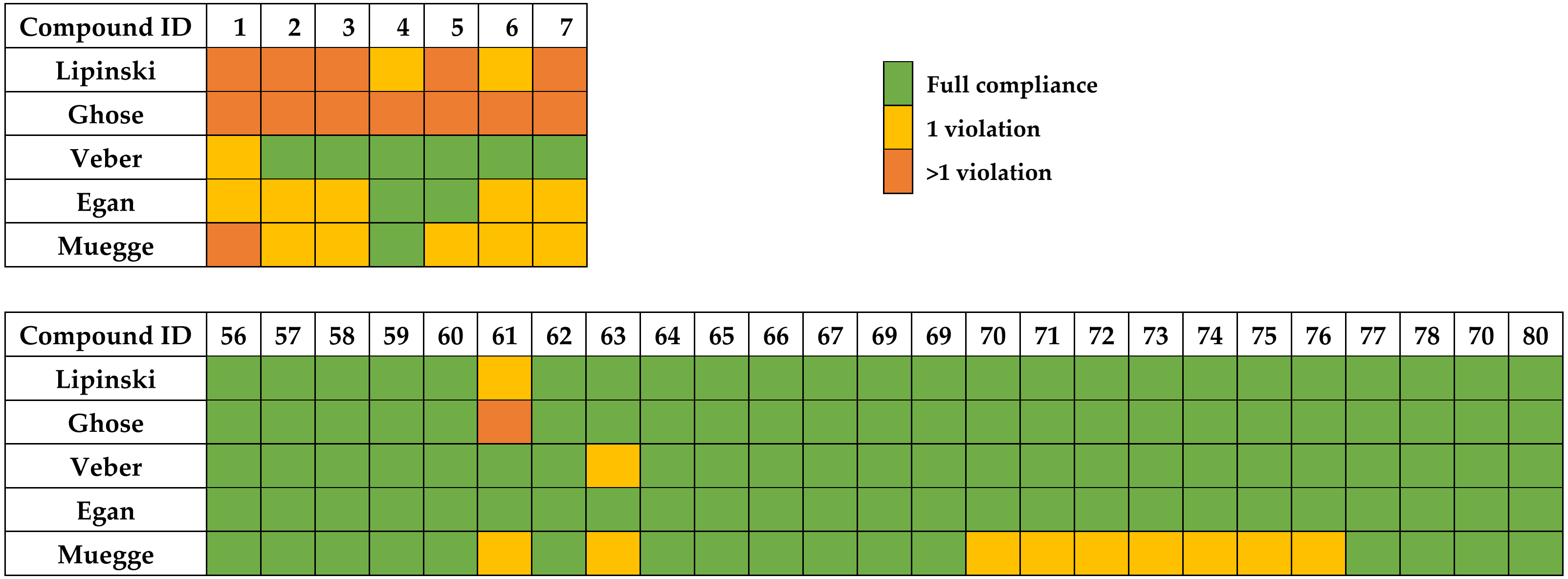

Figure S4. Color map of the compliance with the rules of Medicinal Chemistry. (For interpretation of the references to color in this figure legend. the reader is referred to the Web version of this article.)

Table S6. Predictive pharmacokinetic properties of compounds reported as disruptors of the p53-MDM2 interaction.

	Pharmacokinetic properties							
**Compound ID**	**GI absorption**	**BBB permeant**	**Pgp substrate**	**CYP1A2 inhibitor**	**CYP2C19 inhibitor**	**CYP2C9 inhibitor**	**CYP2D6 inhibitor**	**CYP3A4 inhibitor**
**Selected representative small-molecules targeting MDM2 in clinical trials**								
**1**	Low	No	Yes	No	No	No	Yes	Yes
**2**	Low	No	Yes	No	No	No	No	Yes
**3**	Low	No	Yes	No	Yes	No	No	Yes
**4**	High	No	Yes	No	No	Yes	No	Yes
**5**	High	No	Yes	No	No	No	No	Yes
**6**	Low	No	Yes	No	No	No	No	Yes
**7**	Low	No	Yes	No	No	No	Yes	Yes
**Chalcones reported as disruptors of the p53-MDM2 interaction**								
**56**	High	Yes	No	Yes	Yes	Yes	No	No
**57**	High	Yes	No	Yes	Yes	Yes	No	Yes
**58**	High	Yes	No	No	No	No	No	Yes
**59**	High	Yes	No	No	No	No	No	No
**60**	High	No	No	Yes	Yes	Yes	No	No
**61**	High	No	No	Yes	Yes	Yes	No	No
**62**	High	No	No	No	No	Yes	No	Yes
**63**	High	No	No	No	No	Yes	Yes	Yes
**64**	High	Yes	No	Yes	Yes	Yes	No	Yes
**65**	High	Yes	No	Yes	Yes	No	No	No
**66**	High	Yes	No	Yes	Yes	Yes	No	No
**67**	High	Yes	No	Yes	Yes	Yes	Yes	No
**68**	High	Yes	No	Yes	Yes	Yes	No	Yes

**69**	High	Yes	No	No	Yes	Yes	No	Yes
**70**	High	Yes	No	Yes	No	Yes	Yes	Yes
**71**	High	Yes	No	No	No	Yes	Yes	Yes
**72**	High	No	No	No	No	Yes	No	Yes
**73**	High	Yes	No	No	No	Yes	Yes	Yes
**74**	High	Yes	No	Yes	Yes	Yes	Yes	Yes
**75**	High	Yes	No	Yes	Yes	Yes	Yes	Yes
**76**	High	Yes	No	No	No	Yes	Yes	Yes
**77**	High	Yes	No	Yes	Yes	Yes	Yes	Yes
**78**	High	No	No	No	No	Yes	No	Yes
**79**	High	No	No	No	No	Yes	No	Yes
**80**	High	Yes	No	Yes	Yes	Yes	Yes	Yes

1. Hsu, Y. L.; Kuo, P. L.; Chiang, L. C.; Lin, C. C., Isoliquiritigenin inhibits the proliferation and induces the apoptosis of human non-small cell lung cancer A549 cells. *Clin. Exp. Pharmacol. Physiol.* **2004,** 31, (7), 414-418.
2. Hsu, Y.-L.; Kuo, P.-L.; Lin, C.-C., Isoliquiritigenin induces apoptosis and cell cycle arrest through p53-dependent pathway in Hep G2 cells. *Life Sci.* **2005,** 77, (3), 279-292.
3. Hsu, Y. L.; Chia, C. C.; Chen, P. J.; Huang, S. E.; Huang, S. C.; Kuo, P. L., Shallot and licorice constituent isoliquiritigenin arrests cell cycle progression and induces apoptosis through the induction of ATM/p53 and initiation of the mitochondrial system in human cervical carcinoma HeLa cells. *Mol. Nutr. Food Res.* **2009,** 53, (7), 826-835.
4. Kim, D.-H.; Park, J. E.; Chae, I. G.; Park, G.; Lee, S.; Chun, K.-S., Isoliquiritigenin inhibits the proliferation of human renal carcinoma Caki cells through the ROS-mediated regulation of the Jak2/STAT3 pathway. *Oncology reports* **2017,** 38, (1), 575-583.
5. Tang, Y.; Simoneau, A. R.; Xie, J.; Shahandeh, B.; Zi, X., Effects of the kava chalcone flavokawain A differ in bladder cancer cells with wild-type versus mutant p53. *Cancer Prev. Res.* **2008,** 1, (6), 439-451.
6. Fang, S.-C.; Hsu, C.-L.; Yu, Y.-S.; Yen, G.-C., Cytotoxic effects of new geranyl chalcone derivatives isolated from the leaves of Artocarpus communis in SW 872 human liposarcoma cells. *J. Agric. Food. Chem.* **2008,** 56, (19), 8859-8868.
7. Rao, Y. K.; Kao, T.-Y.; Ko, J.-L.; Tzeng, Y.-M., Chalcone HTMC causes in vitro selective cytotoxicity, cell-cycle G1 phase arrest through p53-dependent pathway in human lung adenocarcinoma A549 cells, and in vivo tumor growth suppression. *Bioorg. Med. Chem. lett.* **2010,** 20, (22), 6508-6512.
8. Lin, E.; Lin, W.-H.; Wang, S.-Y.; Chen, C.-S.; Liao, J.-W.; Chang, H.-W.; Chen, S.-C.; Lin, K.-Y.; Wang, L.; Yang, H.-L., Flavokawain B inhibits growth of human squamous carcinoma cells: Involvement of apoptosis and cell cycle dysregulation in vitro and in vivo. *J. Nutr. Biochem.* **2012,** 23, (4), 368-378.
9. Sławińska-Brych, A.; Zdzisińska, B.; Dmoszyńska-Graniczka, M.; Jeleniewicz, W.; Kurzepa, J.; Gagoś, M.; Stepulak, A., Xanthohumol inhibits the extracellular signal regulated kinase (ERK) signalling pathway and suppresses cell growth of lung adenocarcinoma cells. *Toxicology* **2016,** 357, 65-73.
10. Silva, G.; Marins, M.; Fachin, A. L.; Lee, S. H.; Baek, S. J., Anti-cancer activity of trans-chalcone in osteosarcoma: involvement of Sp1 and p53. *Mol. Carcinog.* **2016,** 55, (10), 1438-1448.
11. Silva, G.; Marins, M.; Chaichanasak, N.; Yoon, Y.; Fachin, A. L.; Pinhanelli, V. C.; Regasini, L. O.; dos Santos, M. B.; Ayusso, G. M.; de Carvalho Marques, B., Trans-chalcone increases p53 activity via DNAJB1/HSP40 induction and CRM1 inhibition. *PloS one* **2018,** 13, (8), e0202263.
12. da Silva Siqueira, E.; Concato, V. M.; Tomiotto-Pellissier, F.; Silva, T. F.; da Silva Bortoleti, B. T.; Gonçalves, M. D.; Costa, I. N.; Junior, W. A. V.; Pavanelli, W. R.; Panis, C., Trans-chalcone induces death by autophagy mediated by p53 up-regulation and β-catenin down-regulation on human hepatocellular carcinoma HuH7. 5 cell line. *Phytomedicine* **2021,** 80, 153373.
13. Lai, C.-K.; Rao, Y. K.; Chang, K.-R.; Lin, C.-W.; Su, H.-L.; Chang, C.-S.; Lai, C.-H.; Tzeng, Y.-M., 3, 3′, 4′, 5′-Tetramethoxychalcone inhibits human oral cancer cell proliferation and migration via p53-mediated mitochondrial-dependent apoptosis. *Anticancer Res.* **2014,** 34, (4), 1811-1819.
14. Qi, Z.; Liu, M.; Liu, Y.; Zhang, M.; Yang, G., Tetramethoxychalcone, a chalcone derivative, suppresses proliferation, blocks cell cycle progression, and induces apoptosis of human ovarian cancer cells. *PloS one* **2014,** 9, (9), e106206.

15. Wang, J.; Yang, Z.; Wen, J.; Ma, F.; Wang, F.; Yu, K.; Tang, M.; Wu, W.; Dong, Y.; Cheng, X., SKLB-M8 induces apoptosis through the AKT/mTOR signaling pathway in melanoma models and inhibits angiogenesis with decrease of ERK1/2 phosphorylation. *J. Pharmacol. Sci.* **2014,** 126, (3), 198-207.
16. Fu, D.-J.; Zhang, S.-Y.; Liu, Y.-C.; Zhang, L.; Liu, J.-J.; Song, J.; Zhao, R.-H.; Li, F.; Sun, H.-H.; Liu, H.-M., Design, synthesis and antiproliferative activity studies of novel dithiocarbamate–chalcone derivates. *Biorg. Med. Chem. Lett.* **2016,** 26, (16), 3918-3922.
17. Iftikhar, S.; Khan, S.; Bilal, A.; Manzoor, S.; Abdullah, M.; Emwas, A.-H.; Sioud, S.; Gao, X.; Chotana, G. A.; Faisal, A., Synthesis and evaluation of modified chalcone based p53 stabilizing agents. *Biorg. Med. Chem. Lett.* **2017,** 27, (17), 4101-4106.
18. Cabral, B. L. S.; da Silva, A. C. G.; de Ávila, R. I.; Cortez, A. P.; Luzin, R. M.; Lião, L. M.; de Souza Gil, E.; Sanz, G.; Vaz, B. G.; Sabino, J. R., A novel chalcone derivative, LQFM064, induces breast cancer cells death via p53, p21, KIT and PDGFRA. *Eur. J. Pharm. Sci.* **2017,** 107, 1-15.
19. Mu, J.; Wang, X.; Dong, L.; Sun, P., Curcumin derivative L6H4 inhibits proliferation and invasion of gastric cancer cell line BGC-823. *J. Cell. Biochem.* **2019,** 120, (1), 1011-1017.
20. Dos Santos, M. B.; Bertholin Anselmo, D.; de Oliveira, J. G.; Jardim-Perassi, B. V.; Alves Monteiro, D.; Silva, G.; Gomes, E.; Lucia Fachin, A.; Marins, M.; de Campos Zuccari, D. A. P., Antiproliferative activity and p53 upregulation effects of chalcones on human breast cancer cells. *J. Enzyme Inhib. Med. Chem.* **2019,** 34, (1), 1093-1099.
21. Salem, M. S.; Hussein, R. A.; El-Sayed, W. M., Substitution at phenyl rings of chalcone and schiff base moieties accounts for their antiproliferative activity. *Anticancer Agents Med. Chem.* **2019,** 19, (5), 620-626.
22. Li, K.; Zhao, S.; Long, J.; Su, J.; Wu, L.; Tao, J.; Zhou, J.; Zhang, J.; Chen, X.; Peng, C., A novel chalcone derivative has antitumor activity in melanoma by inducing DNA damage through the upregulation of ROS products. *Cancer Cell Int.* **2020,** 20, (1), 1-17.
23. Zhang, Y.; Srinivasan, B.; Xing, C.; Lü, J., A new chalcone derivative (E)-3-(4-methoxyphenyl)-2-methyl-1-(3, 4, 5-trimethoxyphenyl) prop-2-en-1-one suppresses prostate cancer involving p53-mediated cell cycle arrests and apoptosis. *Anticancer Res.* **2012,** 32, (9), 3689-3698.
24. Riaz, S.; Iqbal, M.; Ullah, R.; Zahra, R.; Chotana, G. A.; Faisal, A.; Saleem, R. S. Z., Synthesis and evaluation of novel α-substituted chalcones with potent anti-cancer activities and ability to overcome multidrug resistance. *Bioorg. Chem.* **2019,** 87, 123-135.
25. de Vasconcelos, A.; Campos, V. F.; Nedel, F.; Seixas, F. K.; Dellagostin, O. A.; Smith, K. R.; de Pereira, C. M. P.; Stefanello, F. M.; Collares, T.; Barschak, A. G., Cytotoxic and apoptotic effects of chalcone derivatives of 2-acetyl thiophene on human colon adenocarcinoma cells. *Cell Biochem. Funct.* **2013,** 31, (4), 289-297.
26. Fu, D.-J.; Zhang, S.-Y.; Liu, Y.-C.; Yue, X.-X.; Liu, J.-J.; Song, J.; Zhao, R.-H.; Li, F.; Sun, H.-H.; Zhang, Y.-B., Design, synthesis and antiproliferative activity studies of 1, 2, 3-triazole–chalcones. *MedChemComm* **2016,** 7, (8), 1664-1671.
27. Kamal, A.; Dastagiri, D.; Ramaiah, M. J.; Reddy, J. S.; Bharathi, E. V.; Srinivas, C.; Pushpavalli, S.; Pal, D.; Pal-Bhadra, M., Synthesis of imidazothiazole–chalcone derivatives as anticancer and apoptosis inducing agents. *ChemMedChem* **2010,** 5, (11), 1937-1947.
28. Pedrini, F. S.; Chiaradia, L. D.; Licínio, M. A.; De Moraes, A. C. R.; Curta, J. C.; Costa, A.; Mascarello, A.; Creczinsky-Pasa, T. B.; Nunes, R. J.; Yunes, R. A., Induction of apoptosis and cell cycle arrest in L-1210 murine lymphoblastic leukaemia cells by (2E)-3-(2-naphthyl)-1-(3′-methoxy-4′-hydroxy-phenyl)-2-propen-1-one. *J. Pharm. Pharmacol.* **2010,** 62, (9), 1128-1136.

29. Shin, S. Y.; Yong, Y.; Lee, J.; Ahn, S.; Jung, K.-Y.; Koh, D.; Lee, Y. H.; Lim, Y., A novel hydroxymethoxynaphthochalcone induces apoptosis through the p53-dependent caspase-mediated pathway in HCT116 human colon cancer cells. *J. Korean Soc.Appl. Bio. Chem.* **2014,** 57, (4), 413-418.
30. Lee, J. M.; Lee, M. S.; Koh, D.; Lee, Y. H.; Lim, Y.; Shin, S. Y., A new synthetic 2′-hydroxy-2, 4, 6-trimethoxy-5′, 6′-naphthochalcone induces G2/M cell cycle arrest and apoptosis by disrupting the microtubular network of human colon cancer cells. *Cancer lett.* **2014,** 354, (2), 348-354.
31. Singh, N.; Sarkar, J.; Sashidhara, K. V.; Ali, S.; Sinha, S., Anti-tumour activity of a novel coumarin–chalcone hybrid is mediated through intrinsic apoptotic pathway by inducing PUMA and altering Bax/Bcl-2 ratio. *Apoptosis* **2014,** 19, (6), 1017-1028.
32. Loch-Neckel, G.; Bicca, M. A.; Leal, P. C.; Mascarello, A.; Siqueira, J. M.; Calixto, J. B., In vitro and in vivo anti-glioma activity of a chalcone-quinoxaline hybrid. *Eur. J. Med. Chem.* **2015,** 90, 93-100.
33. Shin, S. Y.; Ahn, S.; Koh, D.; Lim, Y., p53-dependent and-independent mechanisms are involved in (E)-1-(2-hydroxyphenyl)-3-(2-methoxynaphthalen-1-yl) prop-2-en-1-one (HMP)-induced apoptosis in HCT116 colon cancer cells. *Biochem. Biophys. Res. Commun.* **2016,** 479, (4), 913-919.
34. Bagul, C.; Rao, G. K.; Makani, V. K. K.; Tamboli, J. R.; Pal-Bhadra, M.; Kamal, A., Synthesis and biological evaluation of chalcone-linked pyrazolo [1, 5-a] pyrimidines as potential anticancer agents. *MedChemComm* **2017,** 8, (9), 1810-1816.
35. Seba, V.; Silva, G.; Santos, M. B. d.; Baek, S. J.; França, S. d. C.; Fachin, A. L.; Regasini, L. O.; Marins, M., Chalcone derivatives 4′-amino-1-naphthyl-chalcone (D14) and 4′-amino-4-methyl-1-naphthyl-chalcone (D15) suppress migration and invasion of osteosarcoma cells mediated by P53 regulating EMT-related genes. *Int. J. Mol. Sci.* **2018,** 19, (9), 2838.
36. Mohamed, M. F.; Hassaneen, H. M.; Abdelhamid, I. A., Cytotoxicity, molecular modeling, cell cycle arrest, and apoptotic induction induced by novel tetrahydro-[1, 2, 4] triazolo [3, 4-a] isoquinoline chalcones. *Eur. J. Med. Chem.* **2018,** 143, 532-541.
37. Wu, L.; Yang, Y.; Wang, Z.; Wu, X.; Su, F.; Li, M.; Jing, X.; Han, C., Design, Synthesis, and Biological Evaluation of Aromatic Amide-Substituted Benzimidazole-Derived Chalcones. The Effect of Upregulating TP53 Protein Expression. *Molecule.* **2020,** 25, (5), 1162.
38. Mohamed, M. F.; Sroor, F. M.; Ibrahim, N. S.; Salem, G. S.; El-Sayed, H. H.; Mahmoud, M. M.; Wagdy, M.-A. M.; Ahmed, A. M.; Mahmoud, A.-A. T.; Ibrahim, S. S., Novel [l, 2, 4] triazolo [3, 4-a] isoquinoline chalcones as new chemotherapeutic agents: Block IAP tyrosine kinase domain and induce both intrinsic and extrinsic pathways of apoptosis. *Invest. new drug.* **2021,** 39, (1), 98-110.
39. Achanta, G.; Modzelewska, A.; Feng, L.; Khan, S. R.; Huang, P., A boronic-chalcone derivative exhibits potent anticancer activity through inhibition of the proteasome. *Mol. Pharmacol.* **2006,** 70, (1), 426-433.
40. Modzelewska, A.; Pettit, C.; Achanta, G.; Davidson, N. E.; Huang, P.; Khan, S. R., Anticancer activities of novel chalcone and bis-chalcone derivatives. *Bioorg. Med. Chem.* **2006,** 14, (10), 3491-3495.
41. Issaenko, O. A.; Amerik, A. Y., Chalcone-based small-molecule inhibitors attenuate malignant phenotype via targeting deubiquitinating enzymes. *Cell Cycle* **2012,** 11, (9), 1804-1817.
42. Selvendiran, K.; Tong, L.; Vishwanath, S.; Bratasz, A.; Trigg, N. J.; Kutala, V. K.; Hideg, K.; Kuppusamy, P., EF24 induces G2/M arrest and apoptosis in cisplatin-resistant human ovarian cancer cells by increasing PTEN expression. *J. Biol. Chem.* **2007,** 282, (39), 28609-28618.

43. Selvendiran, K.; Tong, L.; Bratasz, A.; Kuppusamy, M. L.; Ahmed, S.; Ravi, Y.; Trigg, N. J.; Rivera, B. K.; Kálai, T.; Hideg, K., Anticancer efficacy of a difluorodiarylidenyl piperidone (HO-3867) in human ovarian cancer cells and tumor xenografts. *Mol. Cancer Ther.* **2010**, 1169-1179.
44. Anchoori, R. K.; Khan, S. R.; Sueblinvong, T.; Felthauser, A.; Iizuka, Y.; Gavioli, R.; Destro, F.; Vogel, R. I.; Peng, S.; Roden, R. B., Stressing the ubiquitin-proteasome system without 20S proteolytic inhibition selectively kills cervical cancer cells. *PloS one* **2011,** 6, (8), e23888.
45. Ma, Y.-C.; Wang, Z.-X.; Jin, S.-J.; Zhang, Y.-X.; Hu, G.-Q.; Cui, D.-T.; Wang, J.-S.; Wang, M.; Wang, F.-Q.; Zhao, Z.-J., Dual Inhibition of Topoisomerase II and Tyrosine Kinases by the Novel Bis-Fluoroquinolone Chalcone-Like Derivative HMNE3 in Human Pancreatic Cancer Cells. *PloS one* **2016,** 11, (10), e0162821.
46. Stoll, R.; Renner, C.; Hansen, S.; Palme, S.; Klein, C.; Belling, A.; Zeslawski, W.; Kamionka, M.; Rehm, T.; Mühlhahn, P., Chalcone derivatives antagonize interactions between the human oncoprotein MDM2 and p53. *Biochem.* **2001,** 40, (2), 336-344.
47. Leão, M.; Soares, J.; Gomes, S.; Raimundo, L.; Ramos, H.; Bessa, C.; Queiroz, G.; Domingos, S.; Pinto, M.; Inga, A., Enhanced cytotoxicity of prenylated chalcone against tumour cells via disruption of the p53–MDM2 interaction. *Life Sci.* **2015,** 142, 60-65.
48. Singh, A. K.; Chauhan, S. S.; Singh, S. K.; Verma, V. V.; Singh, A.; Arya, R. K.; Maheshwari, S.; Akhtar, M. S.; Sarkar, J.; Rangnekar, V. M., Dual targeting of MDM2 with a novel small-molecule inhibitor overcomes TRAIL resistance in cancer. *Carcinogenesis* **2016,** 37, (11), 1027-1040.
49. Wu, L.-t.; Jiang, Z.; Shen, J.-j.; Yi, H.; Zhan, Y.-c.; Sha, M.-q.; Wang, Z.; Xue, S.-t.; Li, Z.-r., Design, synthesis and biological evaluation of novel benzimidazole-2-substituted phenyl or pyridine propyl ketene derivatives as antitumour agents. *Eur. J. Med. Chem.* **2016,** 114, 328-336.
50. Brandão, P.; Loureiro, J. B.; Carvalho, S.; Hamadou, M. H.; Cravo, S.; Moreira, J.; Pereira, D.; Palmeira, A.; Pinto, M.; Saraiva, L., Targeting the MDM2-p53 protein-protein interaction with prenylchalcones: Synthesis of a small library and evaluation of potential antitumor activity. *Eur. J. Med. Chem.* **2018,** 156, 711-721.
51. Pereira, D.; Lima, R. T.; Palmeira, A.; Seca, H.; Soares, J.; Gomes, S.; Raimundo, L.; Maciel, C.; Pinto, M.; Sousa, E., Design and synthesis of new inhibitors of p53–MDM2 interaction with a chalcone scaffold. *Arab. J. Chem.* **2019,** 12, (8), 4150-4161.
